# Expression of a Chimeric Antigen Receptor in Multiple Leukocyte Lineages in Transgenic Mice

**DOI:** 10.1371/journal.pone.0140543

**Published:** 2015-10-27

**Authors:** Carmen S. M. Yong, Jennifer A. Westwood, Jan Schröder, Anthony T. Papenfuss, Bianca von Scheidt, Maria Moeller, Christel Devaud, Phillip K. Darcy, Michael H. Kershaw

**Affiliations:** 1 Sir Peter MacCallum Department of Oncology, University of Melbourne, Parkville, Victoria 3010, Australia; 2 Bioinformatics Division, The Walter & Eliza Hall Institute of Medical Research, Parkville, Victoria, 3052, Australia; 3 Bioinformatics and Cancer Genomics, Peter MacCallum Cancer Centre, East Melbourne, Victoria, 3002, Australia; 4 Department of Computing and Information Systems, The University of Melbourne, Parkville, Victoria, 3010, Australia; 5 Department of Medical Biology, The University of Melbourne, Parkville, Victoria, 3010, Australia; 6 INSERM U1043 Centre de Physiopathologie Toulouse Purpan (CPTP), Toulouse, France; 7 Department of Immunology, Monash University, Prahran Victoria 3181 Australia; University of Pittsburgh, UNITED STATES

## Abstract

Genetically modified CD8^+^ T lymphocytes have shown significant anti-tumor effects in the adoptive immunotherapy of cancer, with recent studies highlighting a potential role for a combination of other immune subsets to enhance these results. However, limitations in present genetic modification techniques impose difficulties in our ability to fully explore the potential of various T cell subsets and assess the potential of other leukocytes armed with chimeric antigen receptors (CARs). To address this issue, we generated a transgenic mouse model using a pan-hematopoietic promoter (*vav*) to drive the expression of a CAR specific for a tumor antigen. Here we present a characterization of the immune cell compartment in two unique *vav*-CAR transgenic mice models, Founder 9 (F9) and Founder 38 (F38). We demonstrate the *vav* promoter is indeed capable of driving the expression of a CAR in cells from both myeloid and lymphoid lineage, however the highest level of expression was observed in T lymphocytes from F38 mice. Lymphoid organs in *vav*-CAR mice were smaller and had reduced cell numbers compared to the wild type (WT) controls. Furthermore, the immune composition of F9 mice differed greatly with a significant reduction in lymphocytes found in the thymus, lymph node and spleen of these mice. To gain insight into the altered immune phenotype of F9 mice, we determined the chromosomal integration site of the transgene in both mouse strains using whole genome sequencing (WGS). We demonstrated that compared to the 7 copies found in F38 mice, F9 mice harbored almost 270 copies. These novel *vav-*CAR models provide a ready source of CAR expressing myeloid and lymphoid cells and will aid in facilitating future experiments to delineate the role for other leukocytes for adoptive immunotherapy against cancer.

## Introduction

The generation of transgenic mouse models has become an integral tool in basic fundamental biology for studying the role of a specific gene, and the importance of its function in a single cell right through to the entire organism. In addition to gaining a greater understanding about basic fundamental biology, transgenic mouse models have also been used to generate cellular subsets with useful genetic modifications, facilitating their characterization. This includes fluorescent biomarkers such as the expression of fluorochromes or selectable elements in a certain cellular subset. For example, expression of a gene encoding diphtheria toxin receptor (DTR) and green fluorescent protein (GFP) driven by the Forkhead box p3 (FoxP3) promoter induces transgene expression in regulatory T cells (Treg) and enables their identification by GFP and specific depletion by diphtheria toxin [1]. Similarly, mice bearing transgenes encoding for specific T cell receptor (TCR) genes facilitate the study of T cell biology and their function against defined antigens, such as the OVA-TCR-1 (OT1) and Clone-4 TCR (CL4) systems [2, 3]. These mouse models ultimately become a convenient source for isolating specific cell subsets, eliminating the complex and onerous task of *in vitro* genetic modification of cells. Furthermore, transgene integration is consistent in amount and location, which is often not observed in other methods of genetic modification.

In addition to adding to the basic understanding of the function of a gene, transgenic mouse models have been used to further characterize the expression pattern of specific gene promoters. While the *vav*1 protein (an isoform of *vav*) was known to play a role in immune development and function [4], the transcriptional elements and regulation of the promoter was further revealed through generating a transgenic mouse model whereby the marker transgene β-galactosidase (β-gal), and human CD4 in subsequent studies, was driven by the *vav* promoter [5, 6]. These studies demonstrated the *vav* promoter was capable of driving the expression of a transgene in a transgenic mouse model and, similar to the endogenous *vav*1 protein, transgene expression was restricted to cells of the hematopoietic lineage. Interestingly, they also showed that the expression of the transgene was present during all stages of maturation in multiple immune subsets, indicating the importance of the *vav*1 protein in immune cell development.

The use of transgenic mouse models has greatly added to the wealth of knowledge in the field of immunology and moreover, transgenic mice are often the subject in the development of current therapeutic treatments, particularly in the study of cancer. Adoptive immunotherapy is a promising form of cancer treatment, which involves the activation and expansion of tumor-reactive lymphocytes *in vitro* and their re-infusion into patients [7]. Transgenic mouse models have also played an important role in the optimization of adoptive immunotherapeutic regimens for patients [8, 9].

Adoptive immunotherapy of cancer includes the use of genetically modified T cells with a chimeric antigen receptor (CAR). CAR T cells specific for the CD19 antigen have proven to be clinically efficacious, with recent clinical trials treating a range of blood cancers including B cell acute lymphoblastic leukemia (B-ALL), diffuse large B-cell lymphoma (DLBCL) and chronic lymphocytic leukemia (CLL), achieving up to 90% complete responses in some trials [10–12]. While CD8^+^ T cells have been a major focus in most studies of CAR T cells, recent studies have highlighted the potential for CD4^+^ T cells and macrophages as co-effectors to enhance the anti-tumor effect of adoptively transferred T cells [13–17]. However, very few studies have investigated the therapeutic potential of various other adoptively transferred immune subsets in the context of cancer. Despite the success observed in these studies and the knowledge that the co-transfer of helper immune subsets, together with CD8^+^ T cells, generates a greater anti-tumor response, there have been no studies investigating the anti-tumor potential of different combinations of CAR-expressing leukocyte subsets.

Our minimal understanding of the potential role of CAR-expressing leukocyte subsets stems, at least in part, because of the technical difficulties in genetically modifying many major leukocyte subsets. A common form of stable genetic modification utilizes retroviral vectors, which leads to the integration of the desired transgenes into the genome. Although effective for highly proliferative cells such as T cells, this approach is not yet clinically applicable to more quiescent cells or slower growing cells of the innate immune system. Furthermore, *ex vivo* activation, used to induce proliferation, changes the phenotype of naïve or unstimulated lymphocytes subsets. Finally, as *in vitro* cultured cells often have a short half life, the constant supply of engineered T cells requires new cycles of retroviral transduction be performed regularly, a process that is laborious, costly and time consuming.

To overcome these limitations and study the *in vivo* biology of a range of CAR-expressing immune subsets, we have developed a transgenic mouse model in which the expression of a CAR specific for the human epidermal growth factor receptor 2 (Her2/ErbB2) tumor antigen is driven by the *vav* promoter, which is crucial in immune cell development [18–20] and active in most hematopoietic cells [4]. The CAR was composed of two intracellular signaling chains (CD28 and CD3ζ) linked to an extracellular signaling motif recognizing Her2/ErbB2 [21]. The restricted expression of the *vav* promoter ensured that the expression of the CAR was expressed only on cells of hematopoietic origin [5]. In two different founders, we demonstrate that the *vav* promoter is capable of driving the expression of the CAR on multiple immune subsets, from both lymphoid and myeloid origin. Interestingly, in one of the founders (Founder 9) we observed a very high CAR gene copy number (~270), which was associated with abnormal T cell development and a reduction in T cell numbers in both the thymus and periphery. The second founder (Founder 38) contained fewer integrations [7], whose presence seemed to have less effect on immune development and differentiation. The generation of this transgenic model will further enhance our knowledge about the role of CAR expression in both the development and function of different lymphoid and myeloid subsets.

This work was performed as part of a PhD thesis with publication by C.SM.Yong. Studies characterizing the function and anti-tumor potential of cells derived from these mice will be the subject of a future publication within the same thesis.

## Results

### Generation of the *vav*-CAR transgene and transgenic mice

**Fig 1:**
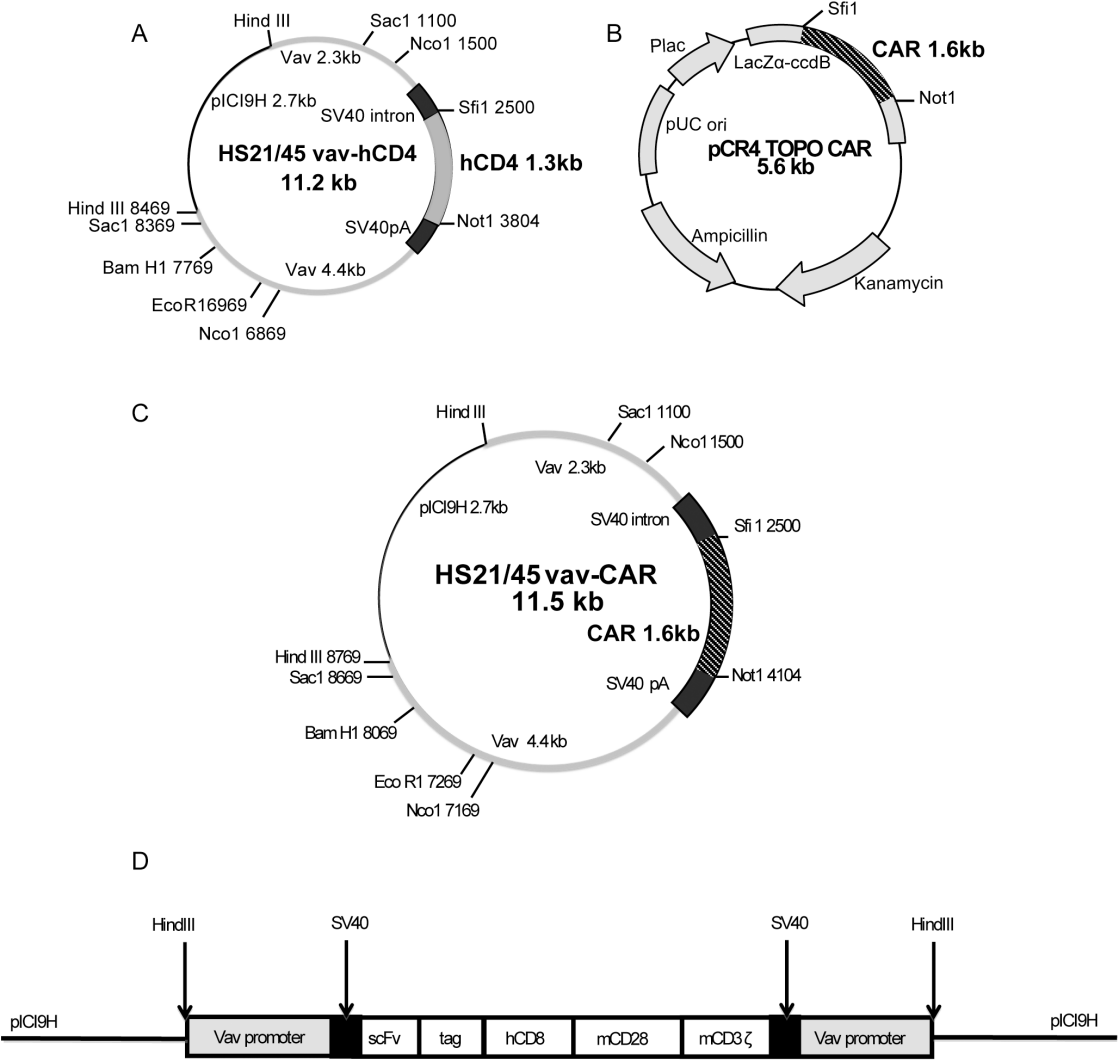
Schematic of *vav*-CAR plasmid generation (A) The HS21/45 plasmid containing the *vav* promoter flanking the transgene human CD4 and (B) the pCR4 TOPO cloning vector containing the chimeric antigen receptor transgene were digested using the restriction enzymes *Not1* and *Sfi1*. The HS21/45 plasmid and the CAR insert were then ligated and subjected to sequencing to verify the correct orientation. (**C**) The *vav*-CAR plasmid was then prepared using an endotoxin free kit, digested with *HindIII* and (**D**) the transgene microinjected into C57BL/6 fertilized oocytes. Not to scale.

Previous work using the HS21/45 promoter in generating transgenic mouse models have reported that the function of the *vav* promoter relies on the presence of the hypersensitivity sites 1, 2, 4 and 5 [6]. The absence of hypersensitivity site 3 was found to have no effect on the function of the promoter. Replacing the first exon in the *vav* gene with a transgene allowed for sufficient expression of the transgene under the control of the *vav* promoter. In our study, we utilized the HS21/45 hCD4 plasmid **(Fig 1A)** and replaced the human truncated CD4 transgene with a chimeric antigen receptor (CAR) that specifically recognizes the human Her2 (ErbB2) antigen **(Fig 1B)**. The *vav* promoter plasmid backbone and the CAR insert were ligated together to generate the *vav*-CAR plasmid in which the *vav* promoter hypersensitivity regions 1, 2, 4 and 5 flank the CAR insert (**Fig 1C and 1D**).

**Fig 2:**
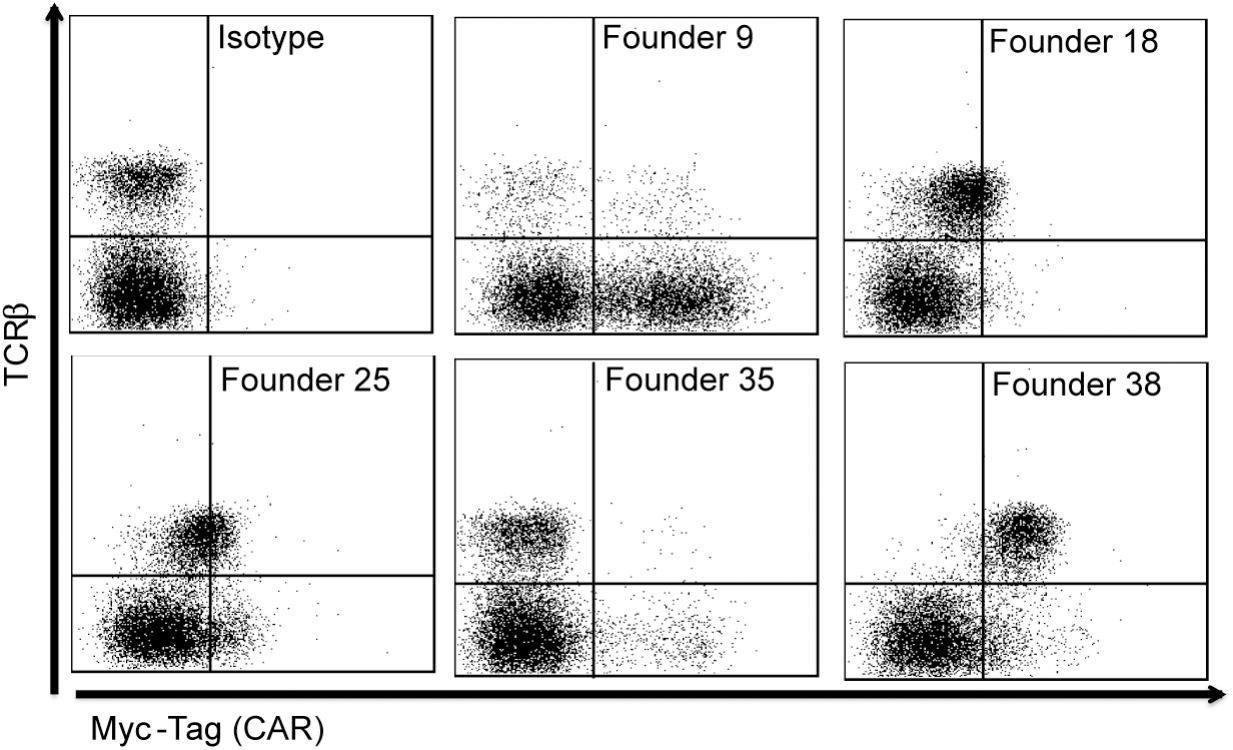
*Vav*-CAR founder mice have varying degrees of CAR expression on peripheral TCRβ^+^ cells. Transgenic pups were tested for the presence of the SV40 sequence by PCR amplification to first identify the presence of the inserted transgene (data not shown). From the primary litter, 5 positive transgenic mice (Founders 9, 18, 25, 35 and 38) were identified out of a total of 43 mice. The level of CAR expression in these mice was then assessed by flow cytometry. Blood was taken from pups in first generation of each founder, erythrocytes were removed and the remaining leukocytes were stained with TCRβ and the Myc-Tag antibody. Representative FACS plots are shown.

To generate transgenic mice, we pronuclear microinjected the digested *vav*-CAR transgene DNA into fertilized oocytes to enable integration of the transgene into the genome. Transgene integration was verified using PCR amplification of the SV40 region (data not shown). Out of 43 pups produced, we identified 5 founder mice positive for the transgene. Each founder was then used to generate an individual *vav*-CAR strain. Transmission of the transgene to following generations was verified by PCR for the 5 founders initially identified. The presence and level of CAR expression on TCRβ^+^ cells was assessed by flow cytometry (**Fig 2**). Phenotypic analysis of the T cell population in each founder strain led us to maintain two strains of the *vav*-CAR population, Founder 9 (F9) and Founder 38 (F38). Founder 9 was chosen based on its distinctly altered immune phenotype as this strain appeared to have a severe reduction in TCRβ^+^ cells compared to both wild type (WT) (data not shown) and F38 mice, with a large proportion of TCRβ^-^ cells expressing the CAR. Founder 38 was chosen as it displayed the highest level of CAR^+^ T cells compared to the other founder strains (**Fig 2**).

### Phenotypic characterization of *vav*-CAR mice

**Fig 3:**
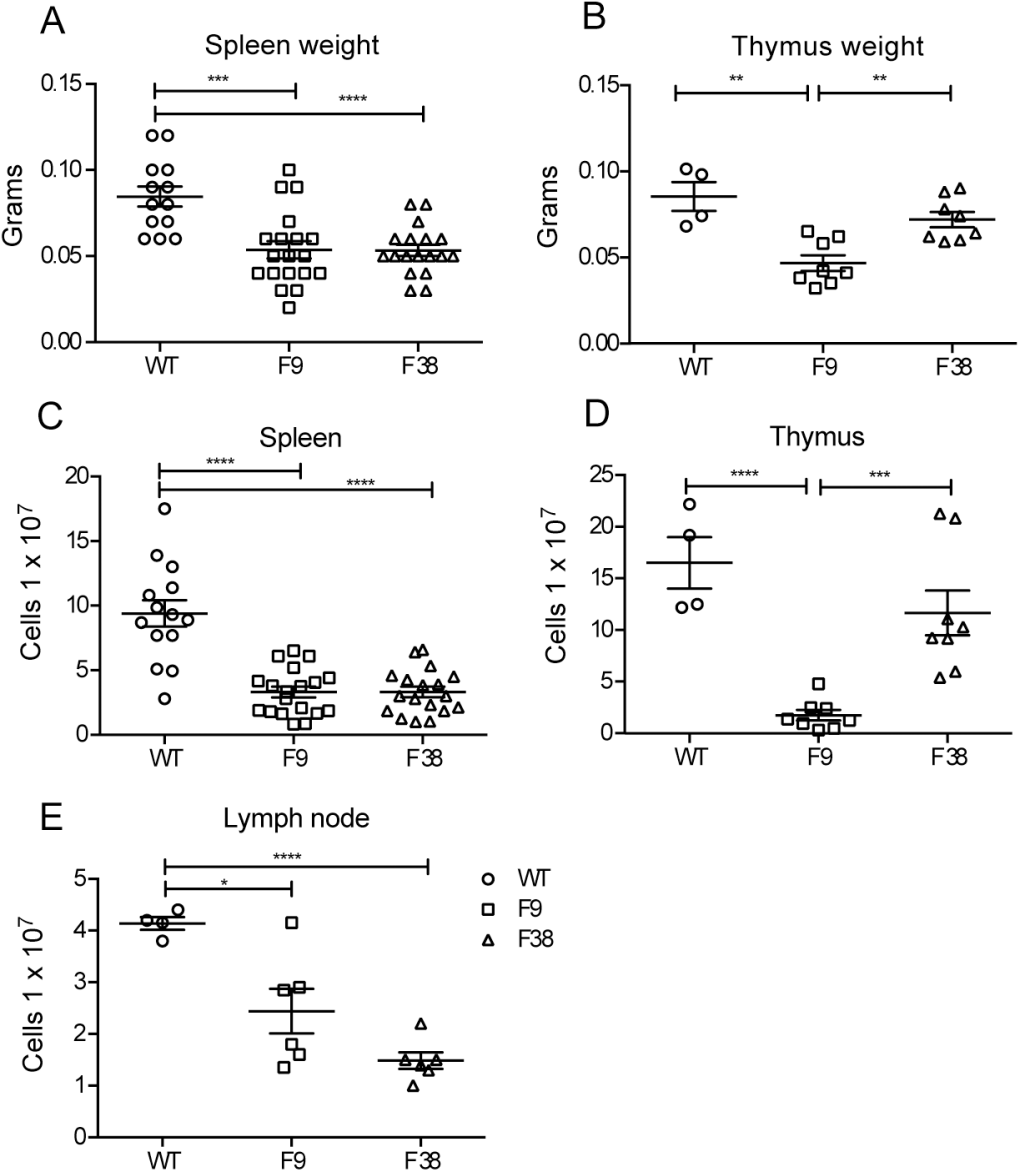
*Vav*-CAR mice have a reduced cellular number in their spleens, thymus and lymph nodes. The **(A, C)** spleens, **(B, D)** thymus and (**E**) inguinal lymph nodes of *vav*-CAR and wild type (WT) mice were harvested and weighed. Organs were dissociated into a single cell suspension and counted. Data represents the mean ± SEM of (Spleen WT = 13, F9 = 19, F38 = 18)(Thymus WT = 4, F9 and F38 = 8)(Lymph node WT = 4, F9 and F38 = 6)) mice. *p = 0.02, **p = 0.001, ***p ≤ 0.0004, ****p < 0.0001.

*Vav*-CAR mice appear anatomically similar to WT mice, with no abnormalities observed in growth, weight or size of these mice between 6 to 12 weeks of age (data not shown). However, the immune related compartments of *vav*-CAR mice were found to be significantly altered compared to WT mice, with a distinct reduction in the weight of the spleen and thymus (**Fig 3A and 3B**). Both F9 and F38 strains were found to have reductions in the cellular numbers observed in multiple lymphoid organs (mean cellular number; spleen of WT 9.4 x 10^7^ ± SEM 1.03e^7^, F9 3.31 x 10^7^ ± SEM 0.41e^7^, F38 3.32 x 10^7^ ± SEM 0.41e^7^) (**Fig 3C**), with the most marked difference observed in the cellular number of the thymus in F9 mice (mean cellular number WT 1.65 x 10^8^ ± SEM 2.48e^7^, F9 1.77 x 10^7^ ± SEM 0.52e^7^, F38 1.17 x 10^8^ ± SEM 2.16e^7^) (**Fig 3C and 3D**). Contrary to both the spleen and thymus, the lymph nodes of F38 mice seemed to have a greater reduction in total cell number than F9 and WT mice (**Fig 3E**).

### *Vav*-CAR mice have an altered immune composition compared to WT mice

**Fig 4:**
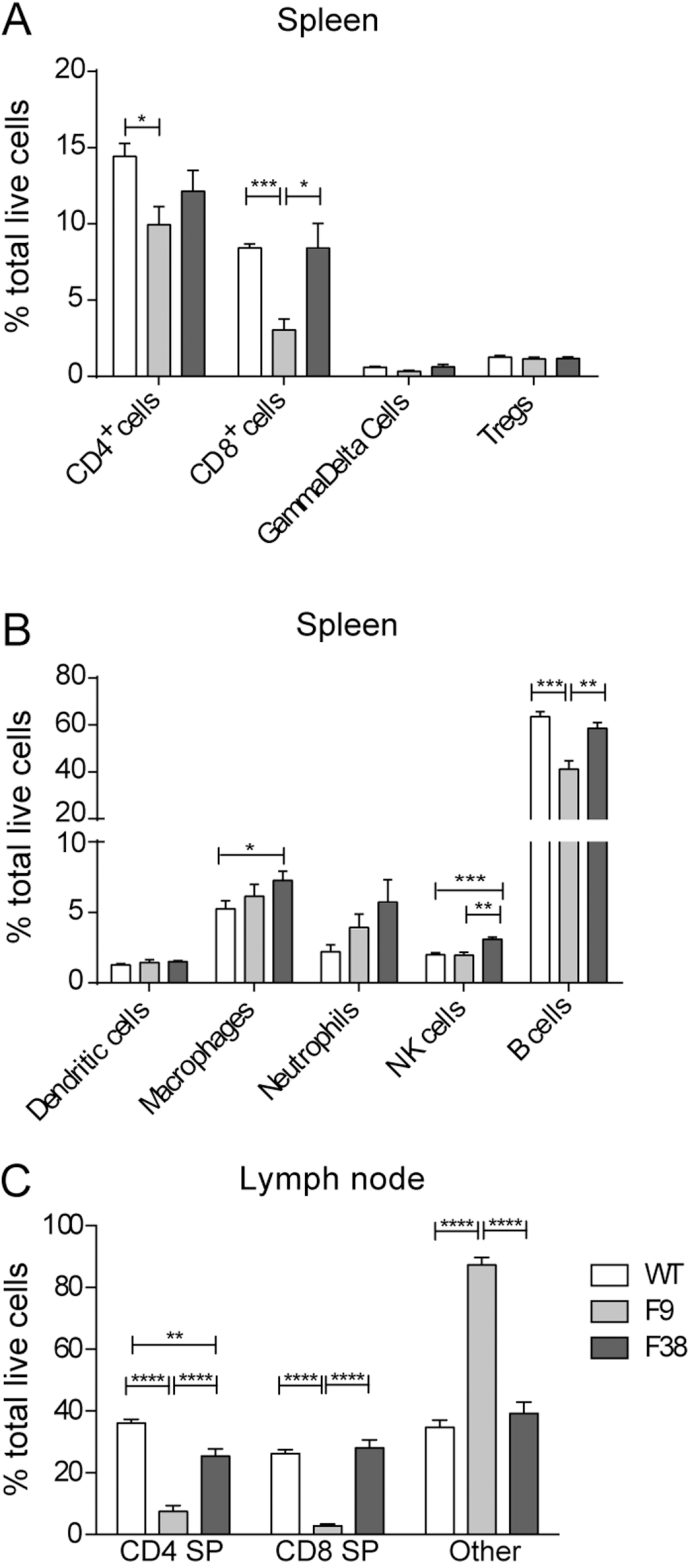
F9 mice display an abnormal immune composition throughout multiple lymphoid organs. The (**A-B**) spleen and (**C**) lymph nodes from *vav*-CAR and WT mice were harvested and dissociated into single cell suspensions. Cells were incubated in Fc receptor block for 10 minutes prior to staining for flow cytometry. 5x10^5^ cells were stained with the following surface markers; TCRβ, CD3, CD4, CD8, CD11C, CD11B, F4/80, GR1, CD19, FR4, CD25, NK1.1, CD49b and γδ (Gamma delta). Data represents the mean ± SEM of (WT = 4, F9 and F38 = 4–8) mice. *p<0.04, **p<0.004, ***p<0.0009, ****p<0.0001. SP: Single positive, Other: Negative for CD4, CD8, CD3 and TCRβ.

To determine whether the reduction in organ size and cellular number was associated with a disruption in the development of the immune system in F9 and F38 mice, we performed flow cytometry analysis to compare the immune composition present in the spleen, thymus and inguinal lymph nodes. We observed a marked difference in the lymphocyte compartment of F9 mice, with a significant reduction in the frequency of single positive CD4^+^ and CD8^+^ T cells and B lymphocytes in the spleen (**Fig 4A and 4B**). However, the myeloid compartment of F9 *vav*-CAR mice was comparable to that of WT mice, with no significant differences observed in the frequency of dendritic cells, macrophages and neutrophils. Interestingly, there was a significant increase in frequency of both macrophages and natural killer cells (NKs) in F38 mice compared to both WT and F9 mice, with almost a 1.5 fold increase in the number of NK cells (**Fig 4B**). This abnormal immunodeficiency in F9 mice was also evident in the lymph nodes, where a significant reduction in the proportion of single positive CD4 and CD8 T cells was apparent (**Fig 4C**). Interestingly, a proportion of splenocytes from F9 mice were lineage-negative (30.85%), lacking expression of extracellular surface markers TCRβ, CD3, CD4, CD8, CD11C, CD11B, F4/80, GR1, CD19, FR4, CD25, NK1.1, CD49b and **γ**δ (**Table 1**).

**Table 1:** Proportion of immune subsets in spleen.

	% total live cells from spleen (mean ± SEM)		
Cell subset	WT	F9	F38
**Dendritic cells**	1.28 ± 0.09	1.43 ± 0.21	1.51 ± 0.07
**Macrophages**	5.25 ± 0.57	6.14 ± 0.85	7.27 ± 0.67
**Neutrophils**	2.22 ± 0.48	3.93 ± 0.96	5.71 ± 1.61
**NK cells**	1.99 ± 0.15	1.96 ±0.22	3.08 ±0.15
**Gamma/Delta T cells**	0.60 ± 0.06	0.33 ± 0.06	0.61 ± 0.16
**Tregs**	1.26 ± 0.12	1.15 ± 0.12	1.18 ± 0.10
**B cells**	63.47 ± 2.19	41.24 ± 3.53	58.64 ± 2.38
**CD4+ T cells**	14.42 ± 0.85	9.95 ±1.19	12.14 ± 1.37
**CD8+ T cells**	8.43 ± 0.25	3.04 ± 0.72	8.40 ± 1.62
**Subtotal (of mean)**	98.92	69.15	98.55
***Other***	1.08	30.85	1.45
**Total**	100.00	100.00	100.00

Splenocytes from *vav*-CAR mice and WT mice were stained with various cell surface markers (markers from **Fig 4**) to determine the proportion of immune subsets. Cells that did not stain positive for any cellular marker were characterized as *other*. Data represents the mean of 4 independent experiments.

### Thymic development is altered in *vav*-CAR mice

**Fig 5:**
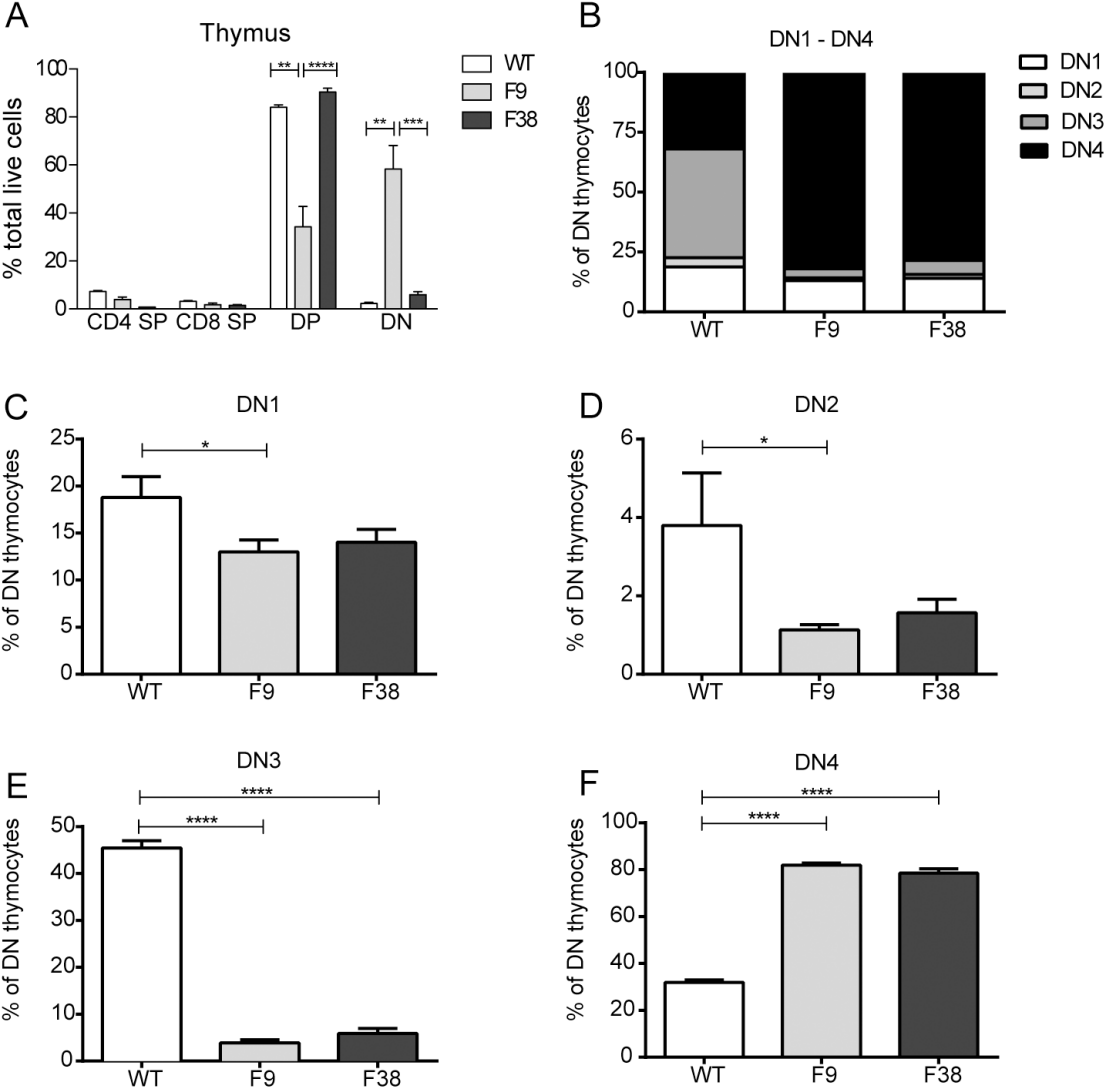
Thymic development is altered in *vav*-CAR mice. The thymus from *vav*-CAR and WT mice were harvested and dissociated into single cell suspensions. Cells were incubated in Fc receptor block for 10 minutes prior to staining for flow cytometry. 5x10^5^ cells were stained with the following surface markers; TCRβ, CD3, CD4, CD8, CD25 and CD44. Data represents the mean ± SEM of **A**; WT = 4, F9 and F38 = 4–8, **B-F** WT = 4, F9 and F38 = 8 mice. *p<0.04, **p<0.004, ***p<0.0009, ****p<0.0001. SP: Single positive for CD4 or CD8, DP: Double positive for both CD4 and CD8, DN: Double negative for both CD4 and CD8, DN1: CD44^+^CD25^-^, DN2: CD44^+^CD25^+^, DN3: CD44^-^CD25^+^, DN4: CD44^-^CD25^-^.

We then wanted to assess whether the reduction in peripheral T cells in F9 mice was due to a defect in thymic development. We observed a significant decrease in the CD4 and CD8 double positive (DP) population and conversely a proportional increase in the double negative (DN) population in the thymus of F9 mice (**Fig 5A**). There seemed to be no alteration in the thymic proportions of F38 mice compared to the WT controls. Despite the major differences in the DN and DP populations between both strains of transgenic mice, we observed a significantly large variance in the development within the double negative population. Indeed, the majority of thymocytes in both strains of *vav*-CAR mice seemed to present in the DN4 stage (**Fig 5B**). Wild type DN thymocytes were observed to be spread almost evenly amongst the DN1, DN3 and DN4 stages of development, sitting at 18, 45 and 32% respectively. The proportion of thymocytes present in both DN1 and DN2 from *vav-*CAR mice was significantly lower than those in WT mice (**Fig 5C and 5D**). More strikingly, only a minority of *vav-*CAR thymocytes were present at the DN3 stage (4% in F9 and 6% in F38), and conversely over 75% present at DN4 (82% for F9 and 79% for F38) (**Fig 5E and 5F**).

### Chimeric antigen receptor expression is present throughout multiple innate and adaptive immune subsets

**Fig 6:**
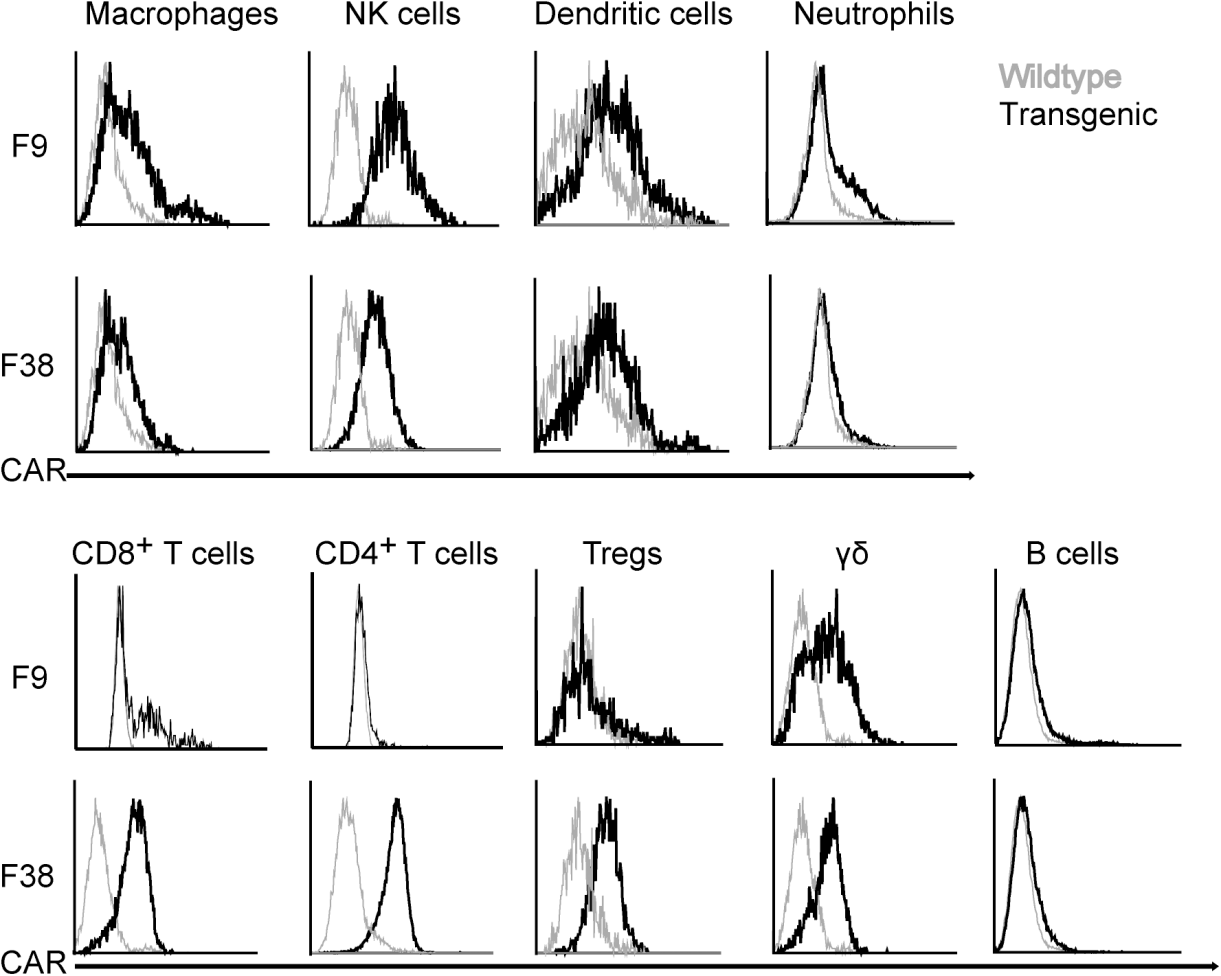
CAR expression is varied between different immune subsets and founders. Splenocytes from *vav-*CAR (black line) and WT (grey line) mice were stained for surface markers (markers from **Fig 4**) and CAR expression. Individual immune subsets were gated on and the level of receptor assessed comparative to WT splenocytes. Representative FACS plot shown for each subset.

**Fig 7:**
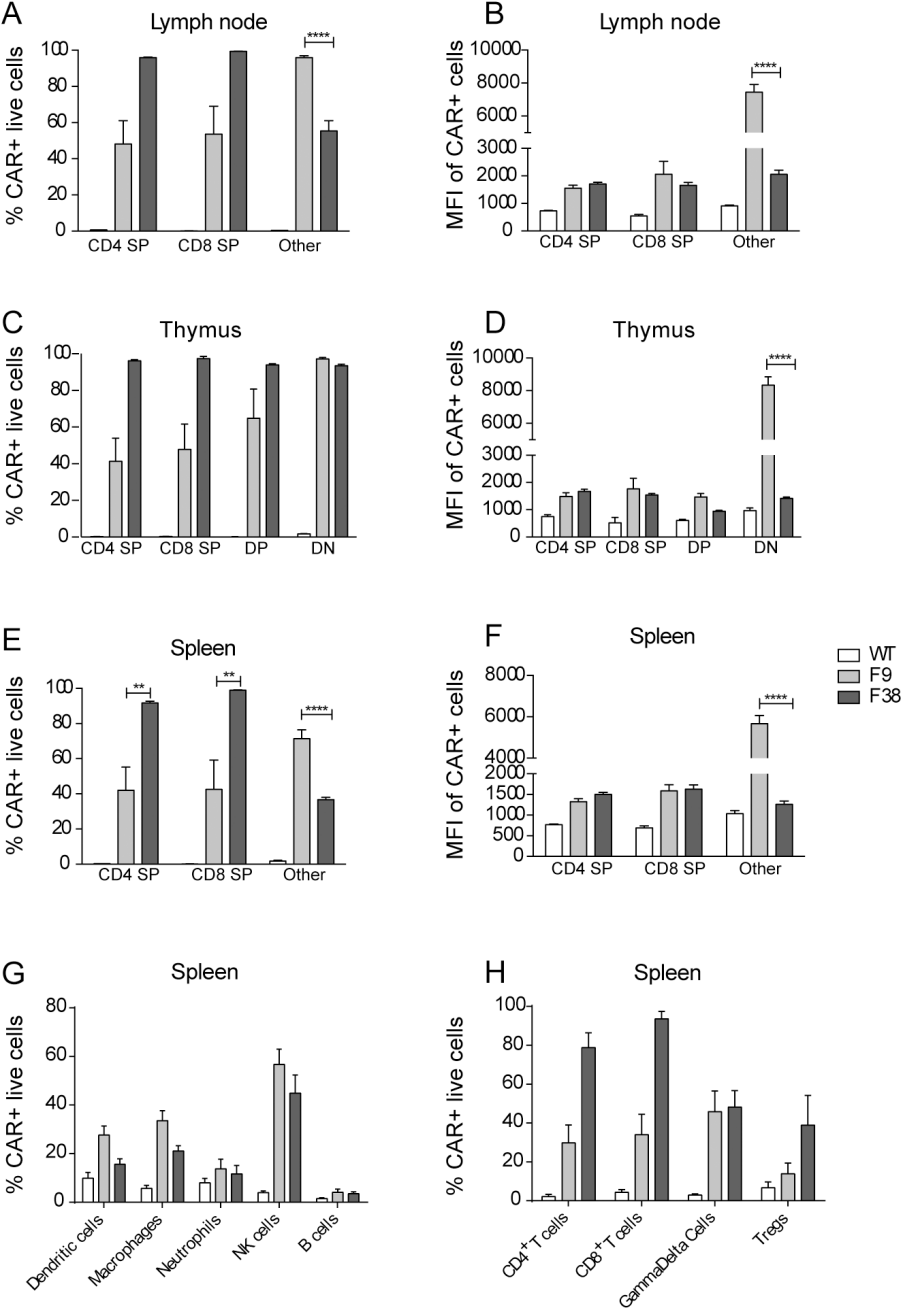
Multiple immune subsets express a CAR in the spleen, thymus and lymph node of *vav*-CAR mice. Splenocytes and lymphocytes derived from the thymus and lymph nodes of *vav*-CAR and WT mice were stained for surface markers (markers from Fig 4) and chimeric antigen receptor expression. (**A, C, E, G, H**) Specific immune subsets were gated on to determine the percentage of CAR positive cells and (**B, D, F**) the mean fluorescence intensity (MFI) of CAR expression. Data represents the mean ± SEM of (WT = 4, F9 and F38 = 4–8) mice. **p<0.007, ****p<0.0001. SP: Single positive, DP: Double positive, DN: Double negative, Other: Negative for CD4, CD8, CD3 and TCRβ.

Previous work using the *vav* promoter in transgenic mouse models had reported high levels of transgene expression in all immune cells, therefore we were interested to investigate this characteristic in our model using a chimeric antigen receptor. Using flow cytometry, we assessed the level of CAR expression in multiple immune subsets from our transgenic model (**Fig 6**). We observed a similar trend within multiple lymphoid organs. The percentage of CAR^+^ immune cells from F9 mice seemed to be consistently 50% of that observed in F38 mice, in all lymphoid organs, however the MFI remained consistent between the two strains (**Fig 7A to 7H**). Almost all T lymphocytes in F38 mice were found to express the CAR, in contrast to those in F9 mice, which had a lower frequency of CAR expression. Intriguingly, there was a lack of CAR expression in B lymphocytes from both founder strains, an observation completely inconsistent with previous studies using the *vav* promoter [5, 6, 22]. Moreover, we observed the lineage-negative population of cells (*other*) from F9 mice (**Table 1**), displayed high levels of CAR expression, significantly more so than any other immune subset in this strain. This high expressing lineage-negative population was observed in the spleen, thymus and inguinal lymph nodes of these mice and consistently expressed higher levels of CAR than the remaining immune subsets (**Fig 7B, 7D and 7F**).

### Mapping the site of transgene integration using whole genome sequencing

**Fig 8:**
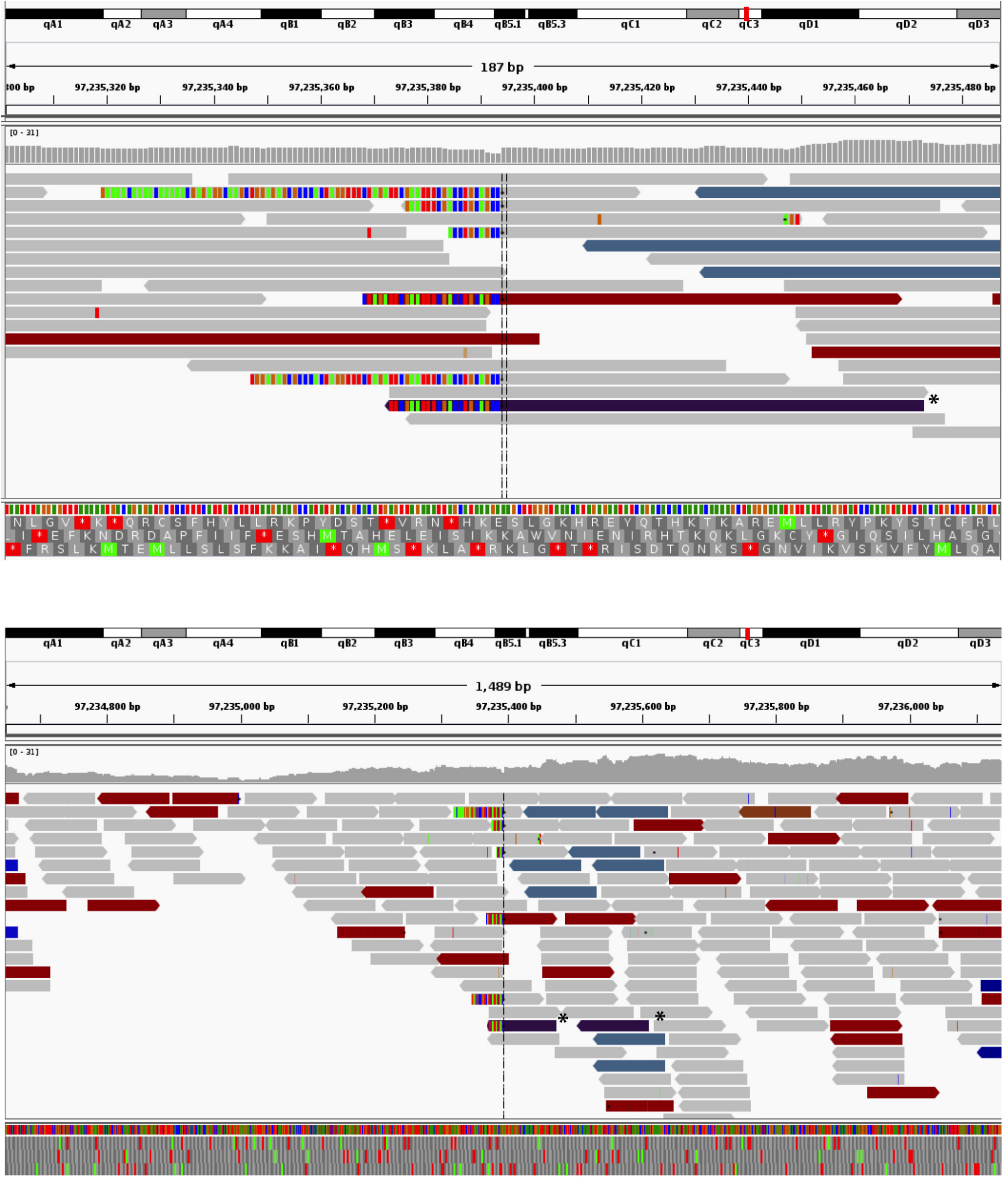
Transgene integration site for F9. Adapted from the screenshots from the IGV genome viewer show the integration sites of the transgene in chromosome 10. The colored reads indicate that the other half of the fragment maps discordantly. The teal colored reads are those that have mates on the transgene, and the purple reads (asterisked) on chr17 (the homologous segments to the transgene material, which are ambiguous for mapping purposes). Multi-colored segments at the ends of reads highlight soft-clipped portions of reads. There is a single cluster of soft-clipped reads for the F9 insertion site.

**Fig 9:**
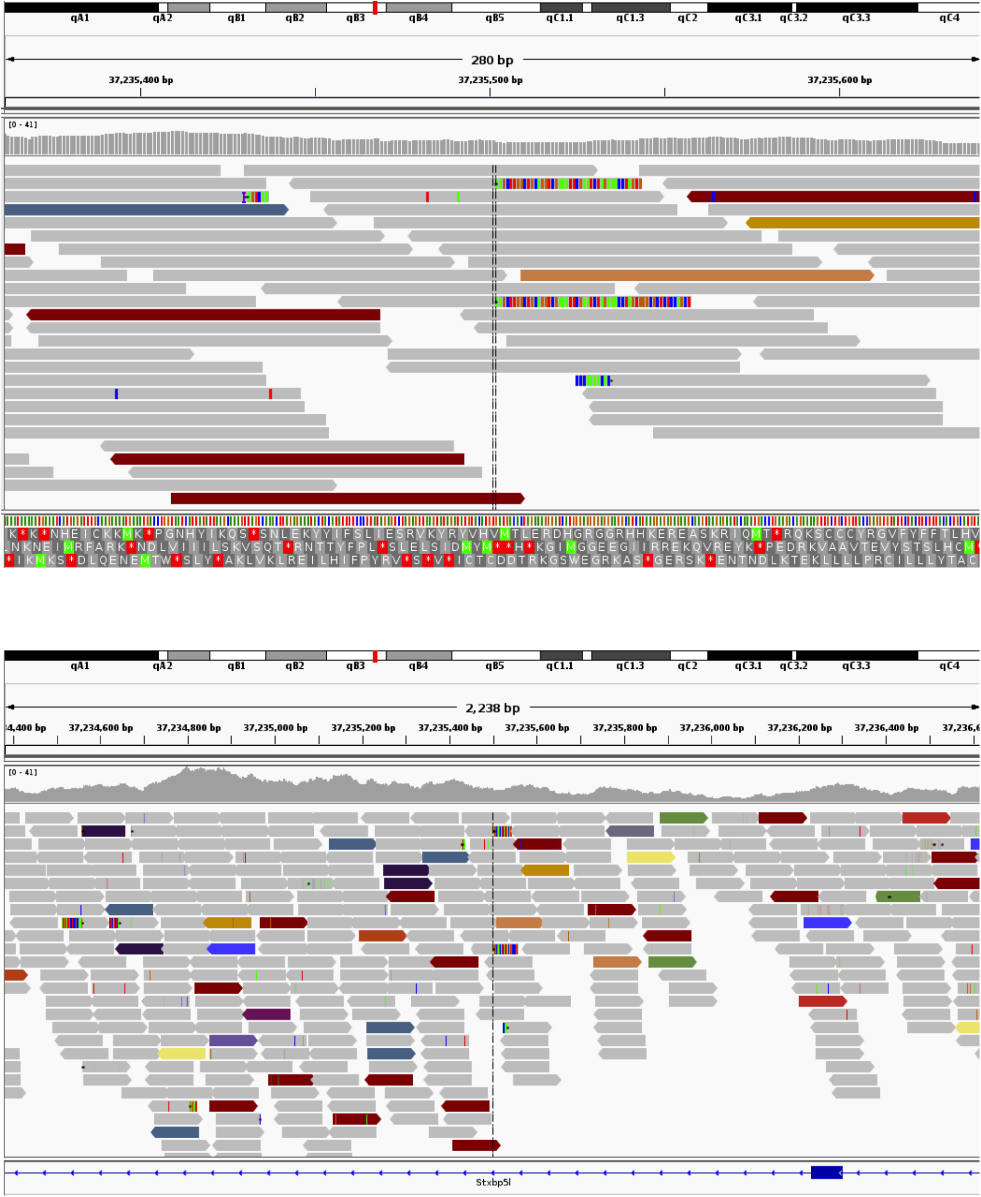
Transgene integration site for F38. Adapted from the screenshots from the IGV genome viewer show the integration sites of the transgene in chromosome 16. The colored reads indicate that the other half of the fragment maps discordantly. The teal colored reads are those that have mates on the transgene, and the purple reads (asterisked) on chr17 (the homologous segments to the transgene material, which are ambiguous for mapping purposes). Multi-colored segments at the ends of reads highlight soft-clipped portions of reads. There is a singular soft-clip for the two sites (entry and exit) of the integration in sample F38.

Given the unusual phenotype of the F9 strain, and the unknown nature of the lineage-negative population present in both the thymus and periphery, we hypothesized that the reduction in lymphocytes in this strain may be due to transgene-mediated interference in the development or maturation of normal T lymphocytes. We decided to utilize whole genome sequencing to determine the site of transgene integration and whether disruption of an endogenous gene essential for T cell development was affected (**Figs 8 and 9**).

From prior knowledge [5, 6, 22] we knew that parts of the transgene were homologous to mm10. An alignment of the putative sequence with Exonerate (version 2.2.0 [23]) established these homologous regions to reside on Chr17:57276770–57279190 and Chr17:57288382–57292241. To derive the exact transgene sequence, we first looked for aberrations in the mappings around those blocks of homology within the native mouse reference. Structural variation detection with Socrates ([24]) detected a total of 86 breakpoints in the sequencing data for F9 (**Fig 8**) and 69 for F38 (**Fig 9**). The breakpoints identified how the transgene components are connected, as well as sub-blocks within the two main components, and an additional segment on Chr17. Further analysis of the read mappings also identified overhanging, non-mapping reads in between the two main components on Chr17. An assembly of those reads (and matching extensions) identified a 2.1 kbp sequence inserted in between the native material. Iteratively, we refined the transgene sequence and identified another short insert of untemplated sequence. The final transgene model had a length of 9038nt and can be reviewed in **S1 Fig.** The transgene was identical in both samples.

We further investigated the predicted breakpoints in each of the samples to establish the insertion sites of the transgene. There were no fusions of the transgene and the native mouse genome predicted by Socrates. We then proceeded to investigate anomalously mapped read pairs and, in particular, fragments that are partially mapped to the transgene and any of the native chromosomes. Clusters of such read pairs indicated the insertion site for both of the samples (**Figs 8 and 9**). Finally, we used Control-FREEC (version 6.7, [25]) for genome wide copy number analysis to establish the number of transgene instances inserted into the DNA. Given the evidence of singular insertion sites in both samples, as well as homozygous fusions from the end of the transgene to its start in both samples we conclude that the multiple copies of the transgene formed a single concatemer, always orientated 5’ to 3’, that has then been integrated into the mouse genome.

**Fig 10:**
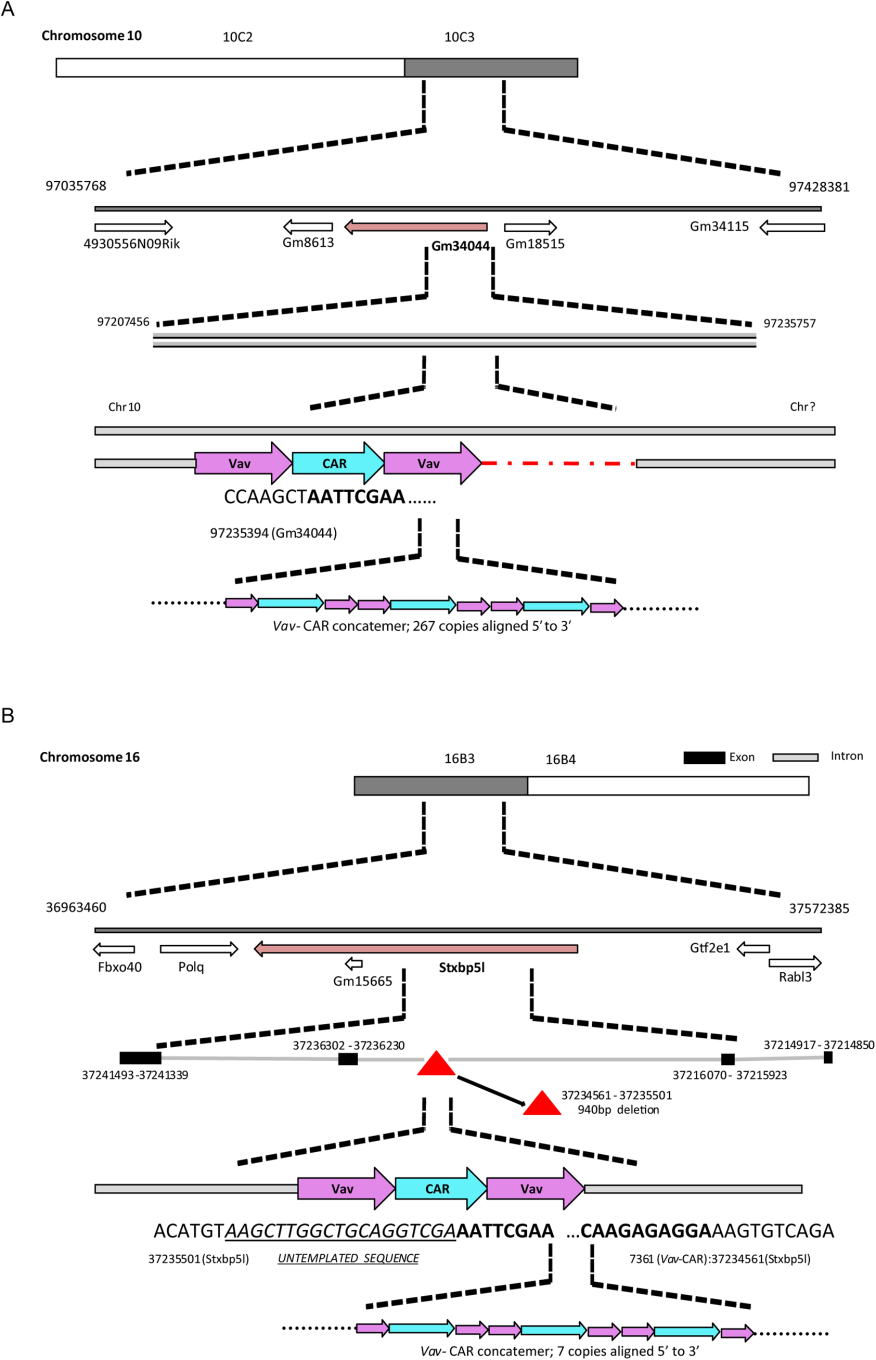
*Vav*-CAR insertion site for F9 (Chr10) and F38 (Chr16) mice. Schematic depicting the transgene integration site for **A)** F9 and **B)** F38 mice. **A)** The entry point for F9 mice was found at position 97235394 on the negative strand on Chromosome 10 within the predicted pseudo gene Gm34044. The exit point for this strain was not found. **B)** The entry point for F38 mice was found at position 37235501 on Chromosome 16 within the Stxbp5l gene. The exit point was found 942bp upstream, at position 37234561, resulting in a 940bp deletion of the Stxbp5l gene. A region of untemplated sequence (underlined) was observed preceding the transgene at the entry point. In both strains, the transgene had formed a concatemer, with **A)** 267 copies in F9 mice and **B)** 7 copies in F38 mice. The fusion position between the native mouse genome (regular font) and the *vav-*CAR transgene (bold) can be seen at nucleotide level.

The results from whole genome sequencing revealed Founder 9 mice harbored a large number of transgene copies, with 267 copies (heterozygotes) integrated in Chromosome 10 at position 97235394 (on the negative strand) (**Fig 10A**). While the 5’ entry point was determined, we were unable to establish the second fusion for F9 that leads back from the transgene into the mouse genome (presumably Chr10). The 5’ insertion point had integrated 363 bp before the 3’ end of the predicted gene Gm34044 (ncRNA XR_380716.1/ LOC102637160) situated at position 97207456 to 97235757. Gm34044 is predicted to be a *linc*RNA (long intervening/long intergenic non-coding RNA), however its function is yet to be characterized. The most proximal gene to the integration site was situated 6.6 kb downstream encoding the predicted pseudo gene Gm18515 (position 97241909 to 97242548).

Founder 38 mice had smaller number of copies, with 7 copies of the CAR transgene integrated at position 37235501 on Chromosome 16 (**Fig 10B**). Surprisingly, the 5’ of the transgene concatemer had integrated almost directly in the middle of another gene, Syntaxin-binding protein 5-like (Stxbp5l) (NCBI gene 207227; start position 37107307 end position 37384985). The entry fusion contained a bit of untemplated sequence in between the transgene and Chr16: AAGCTTGGCTGCAGGTCGA. The exit point lay about 1kbp upstream at Chr16:37234561 and was located at position 7361 on the *Vav-*CAR transgene (over 1.5kbp from the end of the transgene). This resulted in a 941bp duplication of the Stxbp5l gene flanking the transgene insertion site, and placed the transgene in the intron downstream of exon 16 of Stxbp5l. Stxbp5l (also known as tomosyn-2) has been found to play a role in exocytosis regulation of both insulin and acetylcholine, and as such has been nominated as a genetic factor in both type 2 diabetes and neuromuscular disorders [26–29]. Tomosyn-2 (Tom2) knock out mice have been utilized to study its role in neuronal function and regulation and were found to have impaired neuromuscular function and a reduced motor performance. However, there are no reports indicating a role for tomosyn-2 in immune function. No physical abnormalities in terms of development or growth were reported in Tom2^-/-^ mice, and similarly we observed no overt pathologies in F38 homozygous mice.

Despite the process of digesting the transgene DNA prior to injection into the oocytes to create singular linear fragments, the transgene was found to concatenate in a ‘head to tail’ or 5’ to 3’ orientation, forming a long linear fragment of repeating DNA. We observed this in both F9 and F38 mice, with all copies of the transgene found within the genome orientated in a 5’ to 3’ position. While we were able to determine the entry point (5’ end) of the *vav*-CAR transgene into the genome for each founder, we were unable to determine the exit point for F9, possibly indicating the exit point was present in a GC rich or a repetitive region of the genome, and therefore could not be adequately determined, at least with the depth of sequencing used in this study.

### Transgene copy number correlates with both level of CAR expression and proportion of CAR expressing cells

**Fig 11:**
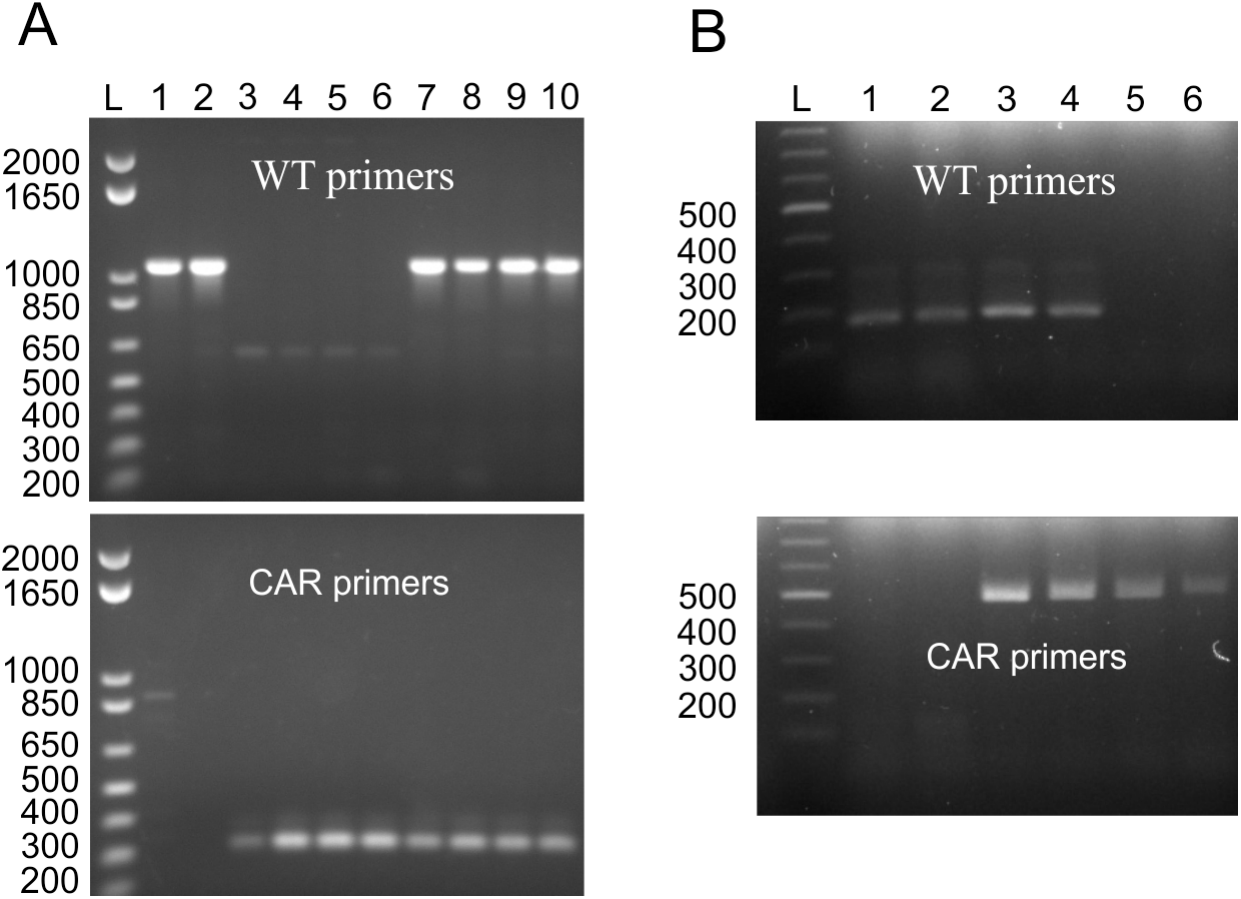
Genotyping of *vav*-CAR mice using PCR primers flanking the transgene insertion site. Genomic DNA from WT, heterozygous and homozygous vav-CAR mice was amplified using primers specific for the endogenous gene flanking the transgene insertion point (WT primers) or within the transgene itself (CAR primers). Amplified DNA was then analyzed on a 1% agarose gel. **(A)** F38 mice, lane L 1 kb Plus DNA ladder, lanes 1–2 wild type C57BL/6 mice, lanes 3–6 homozygous mice, lanes 7–10 heterozygous mice. **(B)** F9 mice, lane L Promega 100 bp ladder, lanes 1–2 wild type C57BL/6 mice, lanes 3–4 heterozygous mice, lanes 5–6 homozygous mice.

We then wanted to adequately validate the genotype of these mice and generate a concise and accurate method to distinguish between homozygous and heterozygous transgenic mice. Previous genotyping had merely relied on the PCR-mediated amplification of the SV40 region present in the receptor (**Fig 1D**). While capable of determining the genotype of WT and CAR mice, it was impractical to determine if there were one or two copies of the integrated allele using this method. Furthermore, we wondered if the homozygosity would affect the level of CAR expression or number of CAR expressing cells in these mice, indeed heterozygous mice might contain a larger proportion of CAR negative cells compared to their homozygous counterparts. Having established the integration site for each founder, we designed PCR primers flanking the integration site as well as within the receptor itself (**S2 Fig**). Using these primers, we confirmed the integration site determined by whole genome sequencing, and moreover adequately identified the zygosity of the *vav*-CAR mice (**Fig 11**).

**Fig 12:**
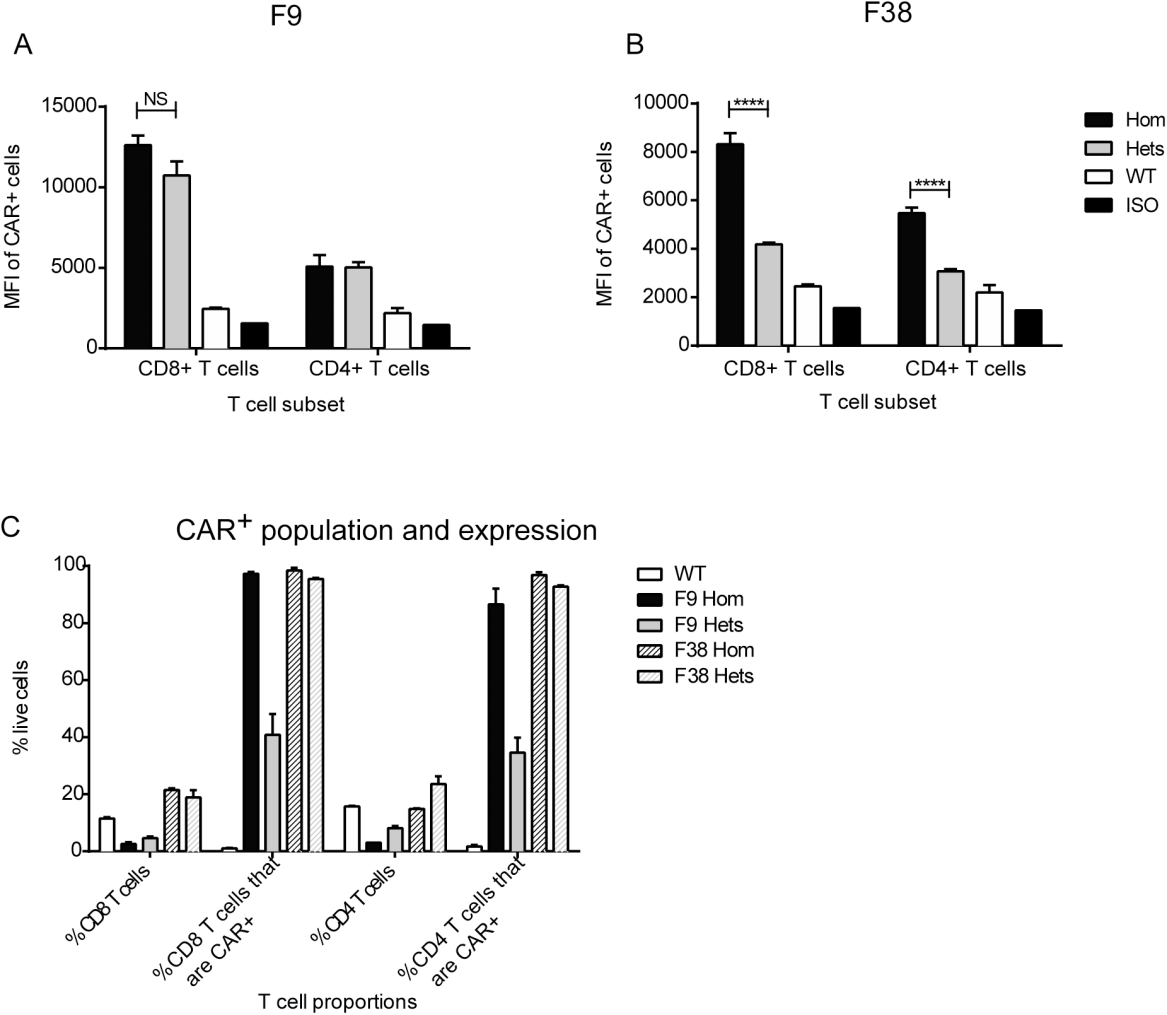
The number of *vav-*CAR transgenes affects both proportion and expression level of CAR^+^ cells. Spleens from homozygous and heterozygous *vav*-CAR mice were harvested and analyzed for the level of receptor expression on resting T lymphocytes. Splenocytes were incubated with Fc receptor block and stained with the following antibodies; TCRβ, CD3, CD4, CD8 and Myc-tag Alexa488. The mean fluorescence intensity (MFI) of CAR expression on F9 mice **(A)** and F38 mice **(B)** is depicted. **(C)** Depicts the percentage of CD4^+^ and CD8^+^ subsets in spleens and the percentage of those subsets expressing CAR in homozygous (Hom) and heterozygous (Het) mice. Data represents the mean ± SEM of (WT = 2, Homozygous = 2, Heterozygotes = 7) individual mice. ****p<0.0001, NS = not significant.

Having established an accurate method to distinguish between heterozygous (het) and homozygous (hom) mice, we then assessed whether transgene copy number was correlative with CAR expression. Interestingly, the number of CAR expressed on cells (as denoted by mean fluorescence intensity (MFI)) and proportion of CAR^+^ cells differed between both heterozygous and homozygous mice from both F9 and F38. We observed no difference in the number of receptors (MFI) from F9 het or hom mice, in both CD4 and CD8 T cells. (**Fig 12A**), yet in contrast, the MFI of CAR expression on F38 het T cells was significantly lower (close to 50% difference) than hom T cells (**Fig 12B**). Interestingly, the gene dosage seemed to have a greater effect in F9 mice on both the proportion of T cells and within that, the proportion of CAR^+^ T cells (**Fig 12C**). Homozygous F9 mice appeared to have a 50% reduction in both CD4^+^ and CD8^+^ T cells compared to heterozygous F9 mice (**Fig 12C**), but of those T cells almost 100% expressed the CAR. Conversely, the proportion of CAR^+^ T cells (both CD4^+^ and CD8^+^), and rather the proportion of T cells in general, from F38 mice was unaffected by the presence of one or two transgene alleles (**Fig 12C**).

### Abnormal T cell development in F9 mice is not due to dysregulated *vav* transcription

The integration site in F9 mice did not appear to occur within a protein-coding gene, let alone a gene essential for T cell development, as we had previously hypothesized. Taken together with the number of transgene copies present, we then hypothesized that the disruption in T lymphocyte numbers and the presence of a T cell marker negative population was perhaps due to the absence of endogenous *vav*1 protein, which is crucial for T cell development [18, 19, 30]. The high copy number of the *vav* promoter transgene in these mice may have provided competition for transcription factors resulting in a greatly reduced transcription efficiency of the endogenous *vav*1 transcript. Consequently, the high level of CAR expression observed in the lineage-negative population may have resulted from the preferential binding of transcriptional elements to the *vav*-CAR promoter, to the detriment of the endogenous *vav*1 gene. In order to validate this, we compared the levels of mRNA transcript of endogenous *vav*1 from thymocytes and splenocytes in both *vav*-CAR mice and WT mice.

**Fig 13:**
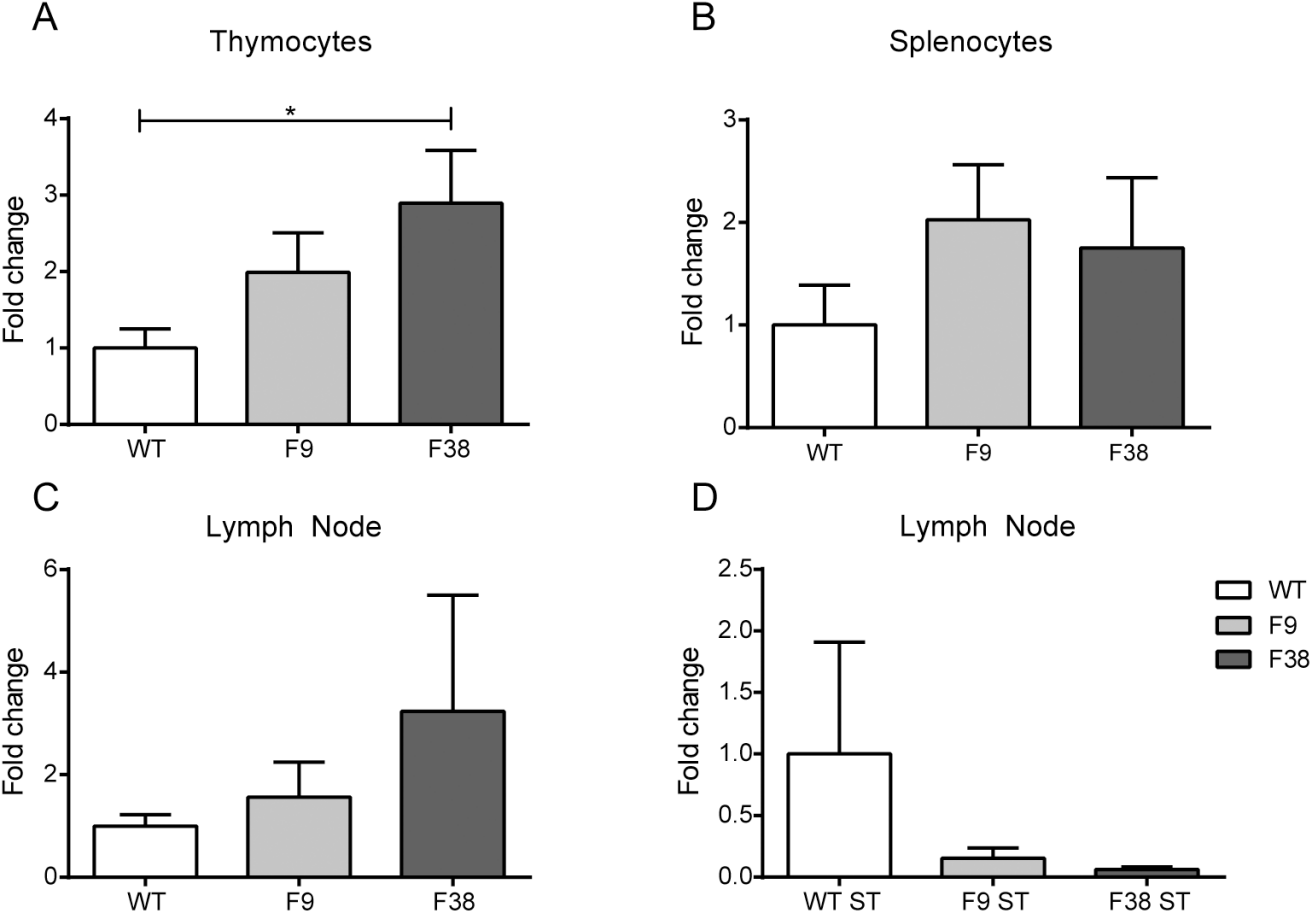
Transcription of *vav* is not altered in *vav-*CAR mice. mRNA was extracted from (**A**) thymocytes, (**B**) splenocytes of 4 week old mice, converted to cDNA and analyzed for endogenous *vav*1 mRNA expression by QRTPCR. *Vav*1 mRNA levels were normalized to the housekeeping gene β-actin. (**C-D**) Lymph nodes were harvested from 6–8 week old mice, dissociated into a single cell suspension and cultured overnight in the (**C**) absence or (**D**) or presence of α-CD3 and α-CD28 antibody stimulation. Cells were harvested the next day, mRNA extracted and converted to cDNA for QRTPCR analysis as above. Data represents the mean fold change in expression ± SEM of triplicate samples from 2–5 individual mice. *p<0.03. ST: stimulated.

We observed no significant decrease in the transcription levels of endogenous *vav*1 in thymocytes and splenocytes from transgenic mice compared to WT mice (**Fig 13A and 13B**). Despite the necessity for *vav* expression and function during thymocyte selection and the large differences between the thymic population of F9 and F38 mice, there seemed to be no altered *vav* expression at this timepoint. As *vav* expression is also known to be integral for TCR downstream signaling pathways ([31, 32]), we also looked at the level of *vav* in peripheral T cells to determine whether the presence of the transgenic *vav* promoters would compromise the ability to upregulate *vav* expression, at least at mRNA level, after stimulation through their TCR. Similarly to the previous results, we observed no differences in the ability of *vav-*CAR T cells to upregulate *vav* expression post-TCR stimulation (**Fig 13C and 13D**). Therefore the high copy number of exogenous *vav* promoters did not affect the transcription of *vav*.

## Discussion

Transgenic mouse models have been an instrumental tool in determining the function and importance of many genes integral to development. Indeed, in addition to answering basic fundamental biological questions, transgenic mice have also been utilized as a tool for generating large numbers of genetically modified cell subsets and have had particular importance in proof of principle studies. In order to better understand the role of immune subsets other than T cells when genetically modified to express a chimeric antigen receptor against a tumor antigen, we took the approach of developing a mouse model in which the expression of a CAR was under the control of an immune specific promoter (*vav*), whose expression is integral in immune cell maturation and development. Here we demonstrate the ability of the *vav* promoter to drive the expression of a CAR transgene in multiple immune cells of both myeloid and lymphoid origin.

Interestingly, we observed a marked difference in both the composition and anatomical appearance of the immune compartment in *vav*-CAR transgenic mice (F9 and F38) compared to WT mice. A distinct reduction in size, weight and cellular numbers from both the spleen and thymus of *vav*-CAR mice was observed when compared to control mice. While both transgenic founder lines were generated in the same manner, through pro-nuclear microinjection, there were evident differences within the immune composition of F9 and F38 mice, suggesting a variation in the integration point of the *vav*-CAR transgene. These differences were apparent in the lymphocyte compartment of F9, whereas the proportion of cells of the myeloid lineage from both F9 and F38 mice were comparable to those from WT mice. The transgene integration of F9 mice had profound effects on the development of T cells, and was apparent during all stages of T cell development as anomalies in the proportion and development of T cells in the thymus, lymph nodes and spleens of these mice were observed.

It is well accepted that the generation of transgenic mice involves many random factors; the number of copies, the orientation of those copies and the integration site itself are all uncontrollable events. While the number of copies differed between F9 and F38 mice, the transgene orientation was similar; in that each transgene copy was orientated 5’ to 3’, creating a long unidirectional fragment of repeating DNA. Linearized DNA used to generate transgenic mice has been reported to form long unidirectional concatemers *in vivo* [33–38]. The linearized DNA is found to re-circularize via intra-molecular ligation, after which homologous recombination between the plasmids results in the formation of a tandem ‘head to tail’ concatemer of transgene DNA. This occurs prior to insertion into the host genome, which is hypothesized to occur via preferential integration based on short homologous sequences at the ends of the introduced concatemer [36]. However, this unidirectional concatenate process is dependent on the concentration of linear DNA injected, with ‘head to head’ or ‘tail to tail’ junctions observed in high concentrations of injected DNA [33]. While amplification of the integrated transgene may also contribute to the large copy number, previous studies have shown a correlative relationship between the integrated copy number and the number of copies injected [33].

Despite finding the entry point for the transgenes, the exit point in F9 mice remains undetermined. While the best scenario is to assume that the transgene has inserted in a linear manner and exited from the genome at the same point of entry (creating a clean break), there also remains the possibility of non-linear insertion, and subsequently the exit point at another location in the genome. Other gene mapping procedures, such as fluorescence in situ hybridization (FISH) or thermal asymmetric interlaced (TAIL) PCR [39], could be used to properly determine the transgene location in the genome and the exact exit point, respectively.

We demonstrated that the *vav* promoter was indeed capable of driving the expression of a CAR throughout multiple immune subsets, from both myeloid and lymphoid lineages in both F9 and F38 mice. The proportion of CAR expressing cells differed between the immune subsets in each mouse strain. The role of *vav*1 in the development and function of T cells and NK cells has been well established [19, 20, 40], however the role of *vav*1 in monocytes is not as well characterized [41]. Studies utilizing *vav*1^*-/-*^ mice have shown it is integral in migration, morphology and complement-mediated phagocytosis in macrophages and is particularly important for integrin regulation in neutrophils [42–45]. Our study also demonstrates that the *vav*1 promoter is transcriptionally active in these subsets as we observed a significant proportion of CAR expressing myeloid cells, albeit the level of CAR expression was lower than those observed in T lymphocytes. This may be due to a reduced necessity of *vav*1 protein for the development and function of these immune subsets compared to lymphoid cells. Indeed, previous studies in *vav*1^*-/-*^ mice have shown that mice deficient in *vav*1 have exhibited altered immune phenotypes, with the most notable abnormalities reported in the development and function of B and T lymphocytes [30, 41, 46]. In addition, our results support the findings that cells of the myeloid lineage may not be entirely reliant on *vav*1 for proper development. While lymphoid development was greatly disturbed in F9 mice, both cellular numbers and proportion of monocytes were comparable to that of WT mice [47]. However, the scope of our study did not properly assess the migratory or phagocytic abilities of monocytes, although it remains possible that these functions may be impaired in F9 mice.

Surprisingly, the results from whole genome sequencing revealed the insertion site in F9 mice did not interrupt a protein-coding gene integral in T cell development, but rather was located within a predicted *linc*RNA (Gm34044), whose function is yet to be characterized. Disruption of this predicted gene may have contributed to the observed phenotype, however we hypothesize that rather, a developmental block at the transition from DN to DP in the thymus due to altered thymic developmental transcriptional networks or possibly early elimination is responsible for the altered immune phenotype observed in these mice.

Given the abnormal phenotype in F9 mice and the developmental block observed in F9 thymocytes, we had hypothesized a reduction in endogenous *vav* transcription may have been responsible. Surprisingly, our data suggested that a reduction in the transcription of endogenous *vav* was not the cause of the developmental block in F9 mice. Previous accounts of *vav*1 deficiency in thymocyte development have observed a large reduction in single positive (SP) and double positive (DP) cell numbers, yet no difference observed in the number of double negative (DN) thymocytes between WT and *vav*1^*-/-*^ mice [18, 19, 48, 49]. We observed in our F9 model similar reductions in both the DP and SP populations, however in contrast to previous accounts in *vav*1^*-/-*^ mice [18, 19] we report a significantly large proportion of DN thymocytes (58% in F9, 2% in WT and 6% in F38).

Studies using *vav* ^-/-^ mice have demonstrated the necessity for *vav* during thymic development, with particular importance during the transition from CD44^-^CD25^+^ (DN3) to CD44^-^CD25^-^ (DN4) in the double negative stage [4, 18, 30, 41, 49–51]. The role for *vav* at this timepoint seems to be integral for the expression of not only the pre-TCR but also for autocrine production of IL-2 [41, 49, 51]. Thymocytes lacking in *vav* are unable to receive adequate IL-2 stimulation, and as a result are arrested in the double negative phase. The thymic phenotype of F9 mice closely mimics that observed in mice with compound deficiencies in both *vav* and an additional gene essential for thymic selection. Developmental blocks between the DN and DP stage have been reported in studies looking at *vav* ^*-/-*^ *Rag-2* ^-/-^ thymocytes [41] as well as *vav* ^*-/-*^ x A1 transgenic TCR mice [18]. Similar observations have also been made in mice deficient in other members involved in the T cell activation pathway. Thymocytes from mice deficient for *slp76*, *lat* or double deficient for *syk/zap70* display a more severe phenotype to F9 mice, with almost 100% thymic arrest in development between the DN3 to DN4 stage [52–54], resulting in a large proportion of DN thymocytes in these models. We had hypothesized the high copy number of transgene *vav* promoters may have diverted transcription factors from the endogenous *vav* promoter, resulting in reduced *vav* transcription and thus causing a developmental block in the thymus in F9 mice. However, we observed no differences in the mRNA levels of *vav*1 in our studies. It is well accepted that transcription factors are promiscuous in nature and often can bind and regulate more than one singular gene. As transcriptional regulation acts as a balanced network, it would be fruitful to widen our RNA analysis, potentially by performing an RNAseq to determine whether other transcriptional programs were affected.

Another hypothesis that may also explain the immunodeficiency observed in F9 mice is that continuous signaling via the intracellular CD3ζ chain of the CAR may have abrogated normal development of T lymphocytes. While both *vav*-CAR mice express the CAR during all stages of thymic development, the differences in the T cell population of these mice may be due to the copy number or number of intracellular CD3ζ molecules present. Previous studies have also observed defects in immune development in transgenic mice bearing high transgene copy numbers. High copy numbers (>11) of an isoform of the major histocompatibility complex class II (MHCII) or the heavy chain constant region locus of B lymphocytes were found to produce defects in B cell development, and similar to F9 mice, a splenic population of “null” cells which did not stain for a number of immune markers [55, 56]. In particular, a transgenic model with a high copy number of the human CD3ε closely mirrors the phenotype we observe in F9 mice, whereby mice containing over 30 copies of the transgene had much smaller thymuses, and were also T cell immunodeficient [57]. Intriguingly, this deficiency was only observed in mice with “high” copy numbers (>10). Similarly, immunodeficiency was only present in our high copy number line (F9) where as immune development in F38 mice, with low copy number, seemed relatively unaltered. Future studies generating transgenic models should take this factor into consideration, that not only the transgene insertion but also the copy number may interrupt normal development.

In addition to the developmental blockade between the DN and DP stages in F9 mice, thymocytes from both transgenic strains seemed to be skewed towards a DN4 stage, with almost 80% of DN thymocytes present at this point of development. Similar observations have been reported in mice overexpressing both the pre-TCRα chain in addition to a pre-rearranged TCRβ chain (pTα Tg^high^ x Vβ5 Tg) [58], with almost all DN thymocytes present in the DN4 stage of development. The excess availability of the pre-TCRα in this model was hypothesized to facilitate a more efficient transition through the DN stages of development, resulting in a higher level of proliferation of DN3 and greater survival of DN4 thymocytes. As *vav* is known to play an integral role in downstream signaling of the pre-TCRα [59, 60], dysregulation in the expression of the *vav* promoter may have potentially contributed to this phenotype. This increase in developmental efficiency may also be due to the internal signaling components of the receptor. The presence of both a CD3ζ and CD28 intracellular signaling chain prior to the expression of the pre-TCRα or TCRβ chain may have provided sufficient stimuli to accelerate the thymic development process, resulting in a much larger population of DN4 thymocytes.

Given the importance of *vav*1 in lymphocytes, it was surprising to note the complete absence of CAR expression in B lymphocytes, an immune subset which had been previously reported to display high levels of transgene expression under the *vav* promoter. In previous *vav* transgenic mouse models, Oglivy *et al*. reported high levels of a human CD4 transgene and the anti-apoptotic protein B cell lymphoma 2 (BCL2) in multiple immune subsets and during all stages of immune development [5, 22]. This information together suggests that the absence of CAR expression in B cells is unlikely to be due to a genetic event, but rather mediated by the CAR itself. Since the transgene used in our model comprised of an extracellular single chain variable fragment of antibody origin, we speculate that recognition of the CAR during the natural negative selection process may have resulted in deletion of CAR expressing B cells, allowing only CAR-negative clones to survive in the periphery. Alternatively, structural elements within the CAR may have rendered it unsuitable for expression in B cells. Furthermore, the CAR transgene encodes a protein encoding immune-signaling elements, unlike previous *vav-*driven transgenes, which may also have contributed to the altered immune phenotypes.

In contrast, we also observed a large proportion of lineage-negative cells present in both the thymus and in the peripheral organs of F9 mice. These ‘*other*’ cells were bereft of most immune surface markers yet expressed the highest level of CAR compared to every other immune subset. The developmental block in DN to DP thymocytes, in conjunction with the high level of receptor expression in these cells, lends support to our hypothesis whereby the high copy number of the *vav-*CAR promoter outcompeted the endogenous *vav* promoter for transcription factors. Indeed, the level of CAR expression on peripheral T cells was proportional to the number of transgenes present (in homozygous or heterozygous mice), and was inversely correlative with the total number of T cells. Based on our findings, we assume these lineage-negative cells are lymphocytes that have not undergone complete development due the reduction in endogenous *vav*1 protein, however we cannot account for how these cells were able to survive the natural selection process in the thymus and furthermore migrate and survive in the periphery. Ironically, while having a detrimental effect on a large proportion of thymocyte development, the presence of receptor and more importantly, its intracellular signaling chains, may have also provided sufficient stimuli for these cells to escape negative selection. There is also the possibility these lineage negative cells may belong to the non-conventional lymphocyte family. The depth of our study did not focus greatly on this arm of the immune system, and other cells such as natural killer T cells (NKTs) [61] or mucosal associated invariant T cells (MAIT) cells [62] may also have contributed to this population. Furthermore, innate lymphoid cells (ILC), a relatively recent addition to this family, are characterized by the lack of conventional immune surface markers [63]. Future work will look into characterizing these *other* cells and assessing their function.

In this study, we introduce a novel mouse model for the study of different immune subsets that are capable of expressing a chimeric antigen receptor. We exploited the expression of the *vav*1 promoter to ensure CAR expression was strictly restricted to immune cell subsets. Two founder mice were generated in this study, Founder 38 with a relatively normal immune phenotype and Founder 9 displaying gross abnormalities in regards to the development and maturation of T lymphocytes. Using whole genome sequencing, we determined the insertion sites and observed a marked difference in the number of transgene insertions in both transgenic mice. While the integration site in these mice had little effect on their natural immune development, it was possible that the copy number of transgenes could interfere with immune development. To this end, we report that the *vav* promoter was indeed capable of driving the expression of a CAR throughout multiple immune subsets in various stages of maturation. A detailed characterization of these mice will greatly benefit future studies investigating the function of CAR expressing immune cells. The use of a transgenic mouse model in which multiple immune cells stably express the same CAR against a tumor antigen will allow for future studies to delineate a role for other immune cells in adoptive immunotherapy for the treatment of cancer.

## Methods

### Generating *vav*-CAR plasmid DNA

Previous studies have described the HS21/45 plasmid containing the *vav* promoter flanking the human CD4 transgene (**Fig 1A**) [5]. The chimeric antigen receptor present in the pCR4 TOPO cloning vector was previously generated in our laboratory (**Fig 1B**) [64, 65]. The chimeric antigen receptor used consists of an extracellular single chain variable fragment specific for the human Her2 (ErbB2) antigen. This is linked to an extracellular c-myc tag and human CD8 hinge region. The intracellular signaling chains consist of mouse CD28 and mouse CD3ζ (**Fig 1D)**. Both HS21/45 plasmid and pCR4 TOPO plasmid were digested using restriction enzymes *Not1* and *Sfi1*. Digested DNA was isolated on a 1% agarose gel. The HS21/45 containing the *vav* promoter and the CAR insert was then extracted and ligated (**Fig 1C**). Nucleotide sequencing was performed to verify the correct ligation (data not shown).

### Generating *vav*-CAR mice

Ethics statement: This study was carried out in strict accordance with the recommendations of the Victorian Bureau of Animal Welfare, Department of Primary Industries, and the National Health and Medical Research Council's Australian code of practice for the care and use of animals for scientific purposes. The protocol was approved by the Institutional Animal Care and Use Committee: Peter MacCallum Cancer Centre Animal Experimentation Ethics Committee under Permit number E498. All efforts were made to minimize suffering. Mice were monitored daily for deterioration in condition and signs of stress, as defined by lethargy, ruffled fur or a hunched appearance, at which time the mice were considered to have reached the ethically permitted humane endpoint criteria and were humanely euthanized. Mice were euthanized using carbon dioxide asphyxiation. Tumors in excess of 150 mm2 were also considered to have reached humane endpoint criteria.

The HS21/45 DNA plasmid containing the CAR insert (*vav*-CAR) was prepared using an Endofree kit (Qiagen, Netherlands) to isolate endotoxin-free DNA. The HS21/45-CAR plasmid was digested using *HindIII* and analyzed on a 1% SeaPlaque Agarose gel (Lonza). The *vav*-CAR transgene was excised, treated with agarase enzyme (New England Biolabs) and centrifuged to pellet residual agarose. The DNA supernatant was then applied to VM Millipore filters (pore size 0.05μM, VMWP02500), suspended in Injection Buffer (10mM Tris (HCl) pH 7.4, 0.1mM EDTA, 10 M NaOH, MilliQ water) and allowed to dialyze in Injection Buffer at room temperature for 5–6 hours. The DNA concentration was assessed by comparing a series of DNA dilutions with a microinjection standard on a 1% gel. The final concentration of DNA was adjusted to 2 mg/ml. The *HindIII* digested fragment encoding the transgene was pronuclear microinjected into fertilized oocytes derived from C57BL/6 mice. Pups were tested for transgene integration by PCR amplification of the SV40 sequence using the following primers SV40 forward 5’-ATGAGTTTGGACAAACCACAACTAG-3’ and reverse 5’-ACCTCCCCCTGAACCTGAAACATAA-3’. Amplified DNA was analyzed on a 1% agarose gel. In all experiments, wild type mice consisted of Her2^+^ transgenic mice [66] or wild type C57BL/6 mice. No difference was observed between both strains in terms of organ weight, cellular number or immune composition for these studies, thus WT mice denote control mice negative for the CAR transgene.

### Flow cytometry

The spleen, thymus and inguinal lymph nodes were harvested from either WT mice, Founder 9 mice or Founder 38 mice and dissociated in phosphate-buffered saline (PBS). Cells were then processed through a 70 μm filter to produce single cell suspensions. Splenocytes and peripheral blood were treated with ammonium chloride potassium (ACK) lysis buffer to eliminate red blood cells. Cells were then counted and resuspended in Fc receptor-blocking buffer (anti-mouse FcγRII/FcγRIII, clone 2.4G2 made in house from tissue culture supernatant) for 10 minutes prior to staining with the following antibodies; TCRβ-PerCP-Cy5.5 (clone H57-597), CD3-e450 (clone 17A2), CD4-APC-eF780 (clone RM4-5), CD8-Pe-Cy7 (clone 53–6.7), CD11C-e450 (clone N418), CD11B-APC (clone M1/70), F4/80-Pe-Cy7 (clone BM8), GR1-APC-eF780 (clone RB6-8C5), CD19-PE (clone eBio1D3), FR4-APC (clone eBio12A5), CD25-PE (clone PC61.5), NK1.1-Pe-Cy7 (clone PK136), CD49b-APC (clone DX5), γδ- Biotin (clone GL3) (Gamma delta), Streptavadin-PE (all from eBioscience), Myc-tag Alexa488 (clone 9B11) (Cell Signaling) or mouse immunoglobulin isotype IgG2a Alexa488 (Invitrogen). All incubations were carried out in the dark on ice. Cells were analyzed on BD FACS CantoII (BD Bioscience).

### Whole Genome Sequencing

Genomic DNA was extracted from the tail of Founder 9 and Founder 38 transgenic mice using the DNeasy Blood and Tissue Kit (Qiagen) according to the manufacturer’s instructions or using phenol:chloroform extraction. The DNA was quantified and the purity verified. 500 ng of DNA were fragmented using a focal acoustic device (Covaris S2) and used to prepare libraries with the KAPA Library Preparation Kit for Illumina platforms (KAPABIOSYSTEMS). Libraries were size selected to an average fragment size of 600 bp using the PippinPrep Instrument (SAGE Science). Three indexed libraries were pooled and sequenced across three lanes of an Illumina HiSeq2500 flowcell using High Output chemistry v3 (Illumina) to generate 2x100 bp reads.

The sequencing data consisted of three lanes each of 100 nucleotides long paired-end (PE) reads generated on an Illumina HiSeq. The F9 data contains about 380 million reads (2*192,120,925) and the F38 data 325 million reads (2*162,782,766). To align the read data we used bowtie2 (version 2.2.3 [67]) and the mm10 build of the mouse genome (http://genome.ucsc.edu) as reference. We ran bowtie2 with the “—local” option to enable it to partially map reads, which is needed for subsequent structural variant analysis. The mapping to the mm10 reference resulted in a 99.6% alignment rate for F9 and an average haploid sequence coverage of 12.8. Similarly, the alignment rate for F38 mice was 99.7% with an average haploid sequence coverage of 10.8. The whole genome sequencing dataset can be found in the European Nucleotide Archive with study title “Expression of a chimeric antigen receptor in multiple leukocyte lineages in transgenic mice”.

### PCR for transgene integration

The following PCR primers were designed to verify the genotype of *vav*-CAR mice. **F9 mice**; Gm34044 forward: 5’-CCATTTGGATTTGCTGTCCT-3’, CAR reverse 5’-TAACCCACCAGATTCCAAGC-3’, Gm34044 reverse: 5’-ATGGGCATTTTCTGTTGGAA-3’. **F38 Mice**; For transgene: Stxbp5l forward 1: 5’-AATGAAATGACCTGGTAATCATT- 3’, CAR reverse: 5’-AGGCGAGGACAATTTCTATCC-3’, For wild-type: Stxbp5l forward 2: 5’-CTAGCAGTTCCTGTTGAGATA- 3’, Stxbp5l reverse: 5’ -ACTTGCTTCTCTCTCCTTATG- 3’. Genomic DNA (gDNA) was isolated from tissue samples of transgenic mice using DNeasy Qiagen Blood and Tissue Kit according to manufacturer’s instructions (Qiagen, Chadstone, Australia). High quality gDNA was required for reproducible PCR of F38 mice. The gDNA was PCR amplified with 35 cycles of 95°C 1 minute, 55°C 1 minute, 72°C 2 minutes, and analyzed on a 1% agarose gel. Alternate primers for genotyping are located in the SV40 poly-adenylation region of the *vav*-CAR transgene: SV40 forward: 5’- ATGAGTTTGGACAAACCACAACTAG-3’, SV40 reverse: 5’- ACCTCCCCCTGAACCTGAAACATAA-3’. The SV40 primers do not allow distinction between heterozygous and homozygous mice.

### Quantitative Real Time PCR

RNA was extracted from thymocytes and splenocytes of 4 week old *vav*-CAR mice and WT mice using the RNeasy Mini Kit (Qiagen) according to manufacturer’s instructions. Inguinal lymph nodes from 6–8 week old *vav-*CAR mice were harvested and dissociated in PBS through a 70 μm filter to form a single cell suspension. Cells were cultured overnight in RPMI plus additives in the presence or absence of anti-mouse CD3 (clone 145-2C11 at 500 ng/ml) and anti-mouse CD28 (clone 37.51 at 500 ng/ml) (BD Bioscience). The cells were harvested the next day and RNA extracted as above. Extracted RNA was converted to cDNA using random primers. Quantitative Real Time PCR was performed on cDNA using primers specific for the *Vav*1 mRNA transcript (Primer code: Hs01041613_m1, Life Technologies). Mouse β-actin primers were used as controls (Primer 4352933E, Life Technologies). The QRTPCR was run according to the manufacturer’s instructions using the StepOne System (Life Technologies)

### Statistics

Statistical significance was determined using an unpaired Student’s T test. P< 0.05 considered to indicate significance difference.

## Supporting Information

S1 Fig *Vav-*CAR sequence found by WGS. Grey highlight indicates flanking regions of the vav promoter. Green depicts the CAR open reading frame, and yellow depicts sequences derived from simian virus 40 (SV40) including a poly adenylation region.
(PDF)

S2 Fig DNA sequence flanking the chromosomal insertion point of the *vav*-CAR transgene in F38 mice. **(Fig A)** Blue highlight = upstream chromosome; Grey highlight = downstream chromosome; Yellow highlight = 5’ end of *vav*-CAR transgene; Green highlight = duplicated section of Stxbp5l gene, flanking seven copies of transgene insertion. Bold underlined text = genotyping primer sequences. **(Fig B)** Schematic representation of the insertion point with PCR primer positions indicated.
(PDF)
