# Supplementary material for: Expression of a Chimeric Antigen Receptor in Multiple Leukocyte Lineages in Transgenic Mice
Source: PLoS One. 2015 Oct 27;10(10):e0140543. doi: 10.1371/journal.pone.0140543 (PMC4624721; doi:10.1371/journal.pone.0140543)
Supplement: S1 Fig — Grey highlight indicates flanking regions of the vav promoter. Green depicts the CAR open reading frame, and yellow depicts sequences derived from simian virus 40 (SV40) including a poly adenylation region. (PDF) [file pone.0140543.s001.pdf]

S1 Fig.

***Vav* promoter**

1 AATTCGAATCTAGAAACCTAGTGGGCGCTCTCCAGCTGCCTCTCGTTTTTGTTTTGTTTT 60

61 CACTGCCTGGCCTTCCTACCCTAACTGCGTGCAAAGAAAACAAATTGTTACCCGGTTTAG 120

121 AGAAGAGGGAGAGGACGCGTAGGAAGTCACAGACGCCAAAAATAGGGGATAGAAATTGTC 180

181 CTCGCCTGTAAGGTTGCAAAACGGGAGGGTTTTCAGGTTGTGCCCACCCCTACCCATATC 240

241 CTCAGTGCGTAAATGAGACTCAAGCTGGTCCAGTGAAGCCGTTTAGCGCCGCCTAGAGGC 300

301 CATAAGGATAAAAGGTGGGGTGCTAAGGAAGCGGCTTGGAATCTGGTGGGTTAGTCAGGG 360

361 AGAGGCTTGCTTGCCAAGGTAATTTTAGTTCCTGAGAAGCTATGGTCAGGAAAGCAGTAC 420

421 ATATTTGTTAGACAAATCAACTAATAAATGACAGGGAAGGGCACTGGTGACCTTGACTCG 480

481 GACGAGAACTCACAGCAGGGAAGGTGGGCTTATAGTATGATAATTAAGGGTGGAGGAGGG 540

541 CTGTGGCTTAAAATTTAGGAAGACTTAAGGGAAAAAAAATGACATTTGGGGGCTGGGGAG 600

601 ATGGTTCAGTCAAATGCTTGCTACTCAAATGAGAGGACAAGAGTTCAAATCCCTAGGGCA 660

661 CAATTAAAGCAGGAGGAGGTAGTCTGTGCTCTTTAATCTCAGTGCTCCTAAGACGAGATG 720

721 GGGGCAGAGACAATTCTGGAAGTTCAGCTAGCCAGGAGCAGTTAGCCCAGGAGTAAGAGA 780

781 GACTCTATGTTAAGTATGTGGAAGAGAGGGTCTACACTGAGGCTGTCCTGCCACCTCAAC 840

841 ACACACGCCATAGTGTTCTCTCTCTCTCTCTCTCTCTCTCTCTCTCTCTCTCTCTCTCTC 900

901 TCTCTTTCTCACACACACACATACAAGTTTTAAAGAAAAAAGGAAAAGTGGGTGGTGAGG 960

961 GTGAGAGGGCTCTGTGGGCAAAGGTGATTGCTACCAAGCCTAATGATCTGAGCTCAATCG 1020

1021 TTGGGGCTTGCATGGTGGAAAGAGGACCTACTCCCCCATGTTAACCTTTGACCTCCACAC 1080

1081 ATGTACTGTGCCCCACATGTACCTTCACACATACACACATACATATGTATATATATACAC 1140

1141 ACACACACATATATATATGTAATAAAAGTGAAATGGCTTTCAAAGTAGAATATATTTTCT 1200

1201 AATGTTTATTATAGTTCTATTTGTTGGCAGTTTCCTACATGTTAACAACCTATCTTCGTC 1260

1261 CTACCCACCCTCAACTCCCCTCTGACTCCGAATGAGAGTGGACTCTCTTTACCTTATCCC 1320

1321 CACTTTCTTGCTCTTTTGTAACCCTTTGCATCCATTACTGCTGCCTGTGGGCATGTTGGC 1380

1381 TCCCTCCTGGCTTTGTCTTGTGCTGCCACCATAACTGCACGGAGTTCATGAGCGTAGTGG 1440

1441 CCATGGGTATGCCCTGGAGATAGCATTTCACATCCCCCGCCCCCGACTCTTACCGACTTT 1500

1501 CTGTTCCTTCTCCCTTGAGCCTCGGGCTGGTGGTGGTATAGATGTCCCATCTATGGCTGA 1560

1561 GCACTCAGTAATCAAAAAGTAACATGTAAGAAGTGATGGAGTGAATTAGTGTCTTCTGTC 1620

1621 AGGTGGTGGGCACAAGTGCAAAGGCCCTGGGGCAGGATGGAGGTCAGGGAGTGAAGAACA 1680

1681 GAGAGAGGAGAAGAGGCTGGCAGGGGTTGGGGCAGGGTGAACAGGGCCTTGTGGGGTGCA 1740

1741 GGAAAGACATGAGTTTTGGCCCCAAAGCAAGGTTGAACCAGGAGGGTTATGGGTGGTGGA 1800

1801 AGAATGGTTGAGACTTGACTCAGTGTTCAGTGGTCCCTTTTGGCTGCTGCAGGGGCAAAA 1860

1861 GGCTGTGAGAGATCAGGGCCGTAGACTGGGGACCAGGAGGAGACAGGAGGTGACAGCCTA 1920

1921 GGTGCCTACGGCTGGATGAGATAGTGGCACCTTAGTTCAGCAGGTGCCCAGGTCTGCTCA 1980

1981 GTGTAGGTGGAGAAAGACGAGGTCACACCAGTGAGTGGAAGCTGTGAGGGCGTAACTGCA 2040

2041 GATGCTGCTCCCCCTCCGCCCCACAGCTCCTCCCCACCCCCACCCCGGGGCGACAGTTAC 2100

2101 AGTCACAGAAGAGGAAGTGGTGTTGTAGTTGTCCCCACTGGGGCAGGCGGCGGCGGTGGT 2160

SV40

2161 GAAGGAACGAGGGTGCACGGGGCCTCCCCGAGGGGCCAAGTGAGAGGCCGGCGCGCCTGA 2220

2221 AGGGGTTCAATTCGAAGTTCCTATTCCGAAGTTCCTATTCTCTAGAAAGTATAGGAACTT 2280

2281 CACGCGTGAACTGAAAAACCAGAAAGTTAACTGGTAAGTTTAGTCTTTTTGTCTTTTATT 2340

2341 TCAGGTCCCGGATCCGGTGGTGGTGCAAATCAAAGAACTGCTCCTCAGTGGATGTTGCCT 2400

2401 TTACTTCTAGGCCTGTACGGAAGTGTTACTTCTGCTCTAAAAGCTGAGATCTGGCCCGTA 2460

Chimeric Antigen Receptor

2461 CGGCCCCACC**ATG**GATTTTCAGGTGCAGATTTTCAGCTTCCTGCTAATCAGTGCCTCAGT 2520
M D F Q V Q I F S F L L I S A S V

2521 CATAATGTCTAGACAGGTACAACTGCAGCAGTCAGGACCTGAACTGAAGAAGCCTGGAGA 2580
I M S R Q V Q L Q Q S G P E L K K P G E

2581 GACAGTCAAGATCTCCTGCAAGGCCTCTGGGTATCCTTTCACAAACTATGGAATGAACTG 2640
T V K I S C K A S G Y P F T N Y G M N W

2641 GGTGAAGCAGGCTCCAGGACAGGGTTTAAAGTGGATGGGCTGGATTAACACCTCCACTGG 2700
V K Q A P G Q G L K W M G W I N T S T G

2701 AGAGTCAACATTTGCTGATGACTTCAAGGGACGGTTTGACTTCTCTTTGGAAACCTCTGC 2760
E S T F A D D F K G R F D F S L E T S A

2761 CAACACTGCCTATTTGCAGATCAACAACCTCAAAAGTGAAGACATGGCTACATATTTCTG 2820
N T A Y L Q I N N L K S E D M A T Y F C

2821 TGCAAGATGGGAGGTTTACCACGGCTACGTTCCTTACTGGGGCCAAGGGACCACGGTCAC 2880
A R W E V Y H G Y V P Y W G Q G T T V T

2881 CGTTTCCTCTGGCGGTGGCGGTTCTGGTGGCGGTGGCTCCGGCGGTGGCGGTTCTGACAT 2940
V S S G G G G S G G G G S G G G G S D I

2941 CCAGCTGACCCAGTCTCACAAATTCCTGTCCACTTCAGTAGGAGACAGGGTCAGCATCAC 3000
Q L T Q S H K F L S T S V G D R V S I T

3001 CTGCAAGGCCAGTCAGGATGTGTATAATGCTGTTGCCTGGTATCAACAGAAACCAGGACA 3060
C K A S Q D V Y N A V A W Y Q Q K P G Q

3061 ATCTCCTAAACTTCTGATTTACTCGGCATCCTCCCGGTACACTGGAGTCCCTTCTCGCTT 3120
S P K L L I Y S A S S R Y T G V P S R F

3121 CACTGGCAGTGGCTCTGGGCCGGATTTCACTTTCACCATCAGCAGTGTGCAGGCTGAAGA 3180
T G S G S G P D F T F T I S S V Q A E D

```
3181 CCTGGCAGTTTATTTCTGTCAGCAACATTTTCGTACTCCATTCACGTTCGGCTCGGGGAC 3240
      L  A  V  Y  F  C  Q  Q  H  F  R  T  P  F  T  F  G  S  G  T

3241 AAAATTGGAGATCGAACAAAAACTCATCTCAGAAGAGGATCTGAATGGGGTCACCGTCTC 3300
      K  L  E  I  E  Q  K  L  I  S  E  E  D  L  N  G  V  T  V  S

3301 TTCAGCGCTGAGCAACTCCATCATGTACTTCAGCCACTTCGTGCCGGTCTTCCTGCCAGC 3360
      S  A  L  S  N  S  I  M  Y  F  S  H  F  V  P  V  F  L  P  A

3361 GAAGCCCACCACGACGCCAGCGCCGCGACCACCAACACCGGCGCCCACCATCGCGTCGCA 3420
      K  P  T  T  T  P  A  P  R  P  P  T  P  A  P  T  I  A  S  H

3421 CCCCCTGTCCCTGCGCCCAGAGGCGTGCCGGCCAGCGGCGGGGGGCGCAGTGCACACGAG 3480
      P  L  S  L  R  P  E  A  C  R  P  A  A  G  G  A  V  H  T  R

3481 GGGGCTGGATCCTAAGCTGTTTTGGGCACTGGTCGTGGTTGCTGGAGTCCTGTTTTGTTA 3540
      G  L  D  P  K  L  F  W  A  L  V  V  V  A  G  V  L  F  C  Y

3541 TGGCTTGCTAGTGACAGTGGCTCTTTGTGTTATCTGGACAAATAGTAGAAGGAACAGACT 3600
      G  L  L  V  T  V  A  L  C  V  I  W  T  N  S  R  R  N  R  L

3601 CCTTCAAAGTGACTACATGAACATGACTCCCCGGAGGCCTGGGCTCACCCGAAAGCCTTA 3660
      L  Q  S  D  Y  M  N  M  T  P  R  R  P  G  L  T  R  K  P  Y

3661 CCAGCCCTACGCCCCTGCCAGAGACTTTGCAGCGTACCGCCCCCTCGAGAGAACAAAATT 3720
      Q  P  Y  A  P  A  R  D  F  A  A  Y  R  P  L  E  R  T  K  F

3721 CAGCAGGAGTGCTGAGACTGCTGCCAACCTGCAGGACCCCAACCAGCTCTACAATGAGCT 3780
      S  R  S  A  E  T  A  A  N  L  Q  D  P  N  Q  L  Y  N  E  L

3781 CAATCTAGGGCGAAGAGAGGAATATGACGTCTTGGAGAAGAAGCGGGCTCGGGATCCAGA 3840
      N  L  G  R  R  E  E  Y  D  V  L  E  K  K  R  A  R  D  P  E

3841 GATGGGAGGCAAACAGCAGAGGAGGAGGAACCCCCAGGAAGGCGTATACAATGCACTGCA 3900
      M  G  G  K  Q  Q  R  R  R  N  P  Q  E  G  V  Y  N  A  L  Q

3901 GAAAGACAAGATGGCAGAAGCCTACAGTGAGATCGGCACAAAAGGCGAGAGGCGGAGAGG 3960
      K  D  K  M  A  E  A  Y  S  E  I  G  T  K  G  E  R  R  R  G

3961 CAAGGGGCACGATGGCCTTTACCAGGGTCTCAGTACTGCCACCAAGGACACCTATGATGC 4020
      K  G  H  D  G  L  Y  Q  G  L  S  T  A  T  K  D  T  Y  D  A
```

SV40

```
4021 CCTGCATATGCAGACCCTGGCCCCTCGCTTTAATTAAGCGGCCGCAGACATGATAAGATA 4080
      L  H  M  Q  T  L  A  P  R  F  N  *

4081 CATTGATGAGTTTGGACAAACCACAACTAGAATGCAGTGAAAAAAATGCTTTATTTGTGA 4140

4141 AATTTGTGATGCTATTGCTTTATTTGTAACCATTATAAGCTGCAATAAACAAGTTAACAA 4200

4201 CAACAATTGCATTCATTTTATGTTTCAGGTTCAGGGGGAGGTGTGGGAGATTTTTTAAAG 4260

4261 CAAGTAAAACCTCGCGAGCCGAAGTTCCTATTCCGAAGTTCCTATTCTACGTAAAGTATA 4320

4321 GGAACTTCGCCCGGGCGGATCCACTAGAGAACCCCTTCGCGCGCCAGCGGATCCACTAGT 4380
```

***Vav* promoter**

```
4381 CACGTGAACTTGCGGCCCCAGATGTCCCAGGTGAGTCTGCTGGGTGATGCTAGGGCCCGA 4440
```

4441 CTGGATAAAGGCTGGTAGGCCCGGCTCTGGCTATGGAGAGGTGTCGAGCAGAACGGAGCT 4500

4501 CTGGGTGGGTTTGTACACTCTTGGTTGGCTTGCAGCTGTCCCAGCAAATTTAGGGGGAAA 4560

4561 TCTCCCTATCCCGCCCCGCCCCCAGCTGACTCTGTTGAGATCCATTTAAAGGGGTCCTAA 4620

4621 GAGGTATCTTGTTGCCTCGTAGTTAGCCAAGAGGGTGCATGAGGTTGGAGAACCAGATTC 4680

4681 AAGAGAGAGAGGCCTTCAGTTTCTCCCCCACCGGTGGCTCCTCCTCTTCCACCTCTTCTG 4740

4741 CCCCCCTCCTCCTCACATCTTCCTTCACCTCCATTTCCTGCCAGGGCTTTGTGCTGTGTG 4800

4801 GCGTGGAGTGTTGGGGGCTGCAATGCGAGACCCTGTGGCATCCCAACTCTACCTATTTCG 4860

4861 GGAAGGGCCCATTGAAGTGCAGGGCCCAGGCCTAGGGTCCTAACAAGGCCCTGGGTGTCC 4920

4921 CTGAGTTTGACATCTGTGCACTCTCCGTGTTTCAAGTGGAGGCATGGTCAGACTATGCCC 4980

4981 CGGTGAGCCTAGAAAATGTATTCAAATCCTTATTTGAATGTGGCTGGGGTGGTGGTGAAA 5040

5041 GGTGACTGGTGGCTTTGTACTCTTTTCTCTGCTTCCCCAGGGATGCTGGGTTTCGGGGGG 5100

5101 AGGGCTGATAGCCCTAGAAATGAAAGGTATAGGTTCAAGTCAAAGGGATCGAGATCTAGA 5160

5161 TATCGATGAATTCGAGCTCGGTACCTGCCGTCATGTATCCACACACAGCCCTTCACTGGT 5220

5221 GGATTCTAGGCAGGGCTCCACCTCTGACCTCGTCCCCAGTCCCTCACTAGTGAATCTAGG 5280

5281 GTAGGGGCTCCACTCAGAGTCACATCCCAGCCCTTAGAGATTTTTATATTTGAGTGGAG 5340

5341 CTGATTGTCAGGAGTCTAGGTTGCACAGCTTTGTGTGTGTCACTGTAGATGTTCGGAGAC 5400

5401 TCTTGGTTGTTTTTTGTTTTTTCAAGACAGGGTTTCTCTGTGTAGCCCTGGCTGTCCTGG 5460

5461 AACTCACTCTGTAGACCAGGCTGGCCTTGAACTCAGAAATCTGCCTGCCTCTGCCTCCCA 5520

5521 AGTGCTGGGATTAAAGGCGTGCGCCATCACGCCCAGCTTAGAGACTCTTTTTTTTAAAGT 5580

5581 GGAAGCACCTGTACCTTGGAAGGTCACCCCGTCCAGCCTTCTCCCATACACTGTCCATTA 5640

5641 CCGCTCTGACTTGCTGTGGCTGCCAGCTGAGCCCCACTTTCCCCTGCACTACCCCACAGC 5700

5701 TGTCCAGTTGTCTGTCCTTGTTACTGTGTACCTCAGCTGTCTGTACTTCCTATCACACAC 5760

5761 TCCAGTTGCTGTTACCAATTGATACCCAGATTGCTTTGTGCTGGAAGCCCCTCCTGTTTA 5820

5821 TCTACCCCACCTCTCCAGGTTATAAGCACTGGGGCATCAGGGTCATGTTTTCCTCACCCA 5880

5881 CTACTGGGTCTCAGGGCTGGGTAAACAGTAAATGCTTAATATATGCTTTTGGAGTCAACT 5940

5941 AATGTCTGGGTTTTTGGTCAGGCTTTGTGTTAGTGCTCAGTTCTAAAGTGGAGAGGTTGA 6000

6001 TTCTCCTTCTCTCTGTTCTTCTGGCTTTCGCCTTCTCTCCCTCTCTCCTTCTTTCCTTTT 6060

6061 CTCCTCTTTCTCTGTTCTTCTTCCAACCCCTCCACATTTGTCCCTCTCCTTCCCTTCCTA 6120

6121 TTCCCTCATCTTTTATCGTATGAATAACATTTTGTCTCCTGGGGGAGATGTGAACACGTG 6180

6181 TCTACTCGTCTCAGACAGGGCCAACAGCCAGGAAAAGCAAGGATCCCGCCAAAGTCCAAC 6240

6241 TTGATGAAGCACTGAGTGTCACTGTGTTACAGATAGGAATGTGGGTCAGGGCTCACTTAA 6300

6301 CAGGAACAGAAATGGCTCAAATACAGCTGTATCACCAAAGCCTGGCATGGATGGTGGGCG 6360

6361 ACTCAGGAAAGCTGCCTTCAGGCAGCTCTGAAAGGTGGACAGTTGTCTCTTCTAGGTGCC 6420

6421 TTGTCCTGTCTGAACCTCTTCTGGGCAGTTCAGCTTGTCTTGAGAGCGGTCACCAGTAGT 6480

6481 CCTTACAGCTTGTGCTTGGGGAGGGCAGTGCCTAGAGAATCTGGTCTGTTTCAGGAACTT 6540

6541 CCTGCATATCTCCTGTTGTTTCCTGAGCTTCACGGGCTTTTCTGTAGGCTAGAATGCTTC 6600

6601 ACCTCCACTTAGCACATCCTATGTCTTAATGAGCTTCCTTCCAAGGTGGAAGGTTTCGTC 6660

6661 TTAAGGGAAATTGCTACACAGCACCCTCTTTCCCCCTCCCTCTGCCTTCTCTCCCTTCCA 6720

6721 CTATCTTCTCCCCTCACCCCTGTTTCCCACCCCTGTTTTTGACACAGGGTCTCACTGTGG 6780

6781 TTCCCTGGACAACCTGGAACTCACTCTGTAGTCCATGCTTACCTCAGAGTTTAGCCATCC 6840

6841 TTTGCTCTGCCTCCTCTTCCTGAGCGCGGAGATTACAGGTATATTCCACCACACTGGGAT 6900

6901 TTCATTTCTGATTTTTGGTTGGAGAACCCAAGGCAGGATGACCTGTCCAAGGTCCCCCTG 6960

6961 CTTACCAGTTTCAGCTGGGAGTCACCAGACACCTAGTTGTGGAAGGCGTACTGGGGTGTG 7020

7021 AGGTGACAGGGAAAGAAGAGGAAGTCTGATTCCCAGAATGCTTTAGACTCATGCTGAAGA 7080

7081 GTTGGGTCATCAGCTCCTCAATTCAAAGCTCTGGACAGGATGAGCAGCGTGACCACCCGC 7140

7141 TCTGGGAAGCCTCAGGGAGCTAGCTGGAGTCTGAAAGATTACAGGGCTGAAGGAAGGCAG 7200

7201 AGAGCCATGCCTCTACCCCTTGTCTATCTTCCCCAGCCAGGCCTGGTAACAGGCATTTCC 7260

7261 TGTTTCCGGCCATCTCCTGGGTTTGGGTTGTGGGTGTGGAAAGCGCAGAAGGCAGAATTT 7320

7321 GTGTTGAGTCTGGGCCGCTTTCTCCCAGCCCCCAAGAGAGGCCACTGTCCTTACTGCTAC 7380

7381 TTCCTTGAACAGAATTCTCTCTATTTTGGGCAGAGGCACCAAAAGCCAACTTCCGGCTTG 7440

7441 ATCACAGTGCCTGGCTCCTGATTTGCCTAAAGCCTTCTTGCTTGTCTAGCGCCATGGTCT 7500

7501 TCTTTCGTCCCGGAGGATAAGGGAAGCAGAGGGGTTGTCCCTATAAAACAAGGAAGAATT 7560

7561 CTCCTTCCACGTGCCTCCTACAGTGAGTGGCTTCCTGTCCTTCATCATCTTTTCACATGC 7620

7621 CTTACGGTGCCAGGAAGTTCTTCTGGTGGTCTAACCACAGTCACCGTGCTTTCTGAAGGA 7680

7681 GCCCAGCTCTCTAACTAGCTACTCTGAAGCGGTGGAAGGGGTTATGAATCATACTCCTTT 7740

7741 TGGAAGAGTCATGTGACTTGAGGGAAGGCAGTCTCAAAGCTCAGCTGATTCTCTGAGGTT 7800

7801 AGGAGGGGGATTTCCTTCCTTAGGCCACACAGACCTAAGGCCAGACATCACCTCTGGTGT 7860

7861 TGGTCTGCCAGCCCTGTCTGCCTGTCTATGGCCCACAGGACTTCTGAGTGCTGTATATAA 7920

7921 TGGTGTATATAATTTCTGTGATATGCTGGTCCCTGTTATGTCCCCGTGAGGCCTGGGCGA 7980

7981 GGGGGCTGTGGTGGCTCAATGTGGTTCAGGAGCTAGGGTAGAGCAGGAGTGGGGCTGGGT 8040

8041 TCTGTGTGTTTCTCCACACTCCTGCTTTTACATGTGTGACTCTGGCAGGTGACAGCCCTG 8100

8101 TGACAAGCCTCAGTTTTCCCACCTGTCAGATAGAAGTCCTTTGTTTAGCCATTTCGTCTT 8160

8161 TCACTTGTGCGTGTGTGTATTTGTATGCATTTGTGCATGTTTATTATTACTATTATATTG 8220

8221 TGTGTGTGTGTGTGTGTGTGTGTATGGGTGCAGATGCCTGAGGAGGCCAGAAGAGGGCAT 8280

8281 TGGATCCTCCTGAGTTGGACTCACAAGTGTGTGTGATATGGGATGGAAACCAAACTTGAC 8340

8341 TCCTCTGTGAGATGGACAGTGCCAGCACCCCTGAACATTCTCGTGGGTTTAAGCATTGAA 8400

8401 CCCAGGGCCTTGCTTTAGCAATTGAGTTGCCTCTCAGACCTCCTTCAATAACTTAGAAAT 8460

8461 TAGAGCATATTTTTTTTTTGGCAGTTTGGTCTGTGTACATATGTATTTTGATCATGGCCG 8520

8521 CTCTACTCCCTTGTCTTTTTTGTGTTGTTGTTGTTGTTGTTTTTTTTTTTTTGTGTGACC 8580

8581 CAGTGAGTTGAATTAGAGTTTGTGACAGGAGTCAGGGTGAGGGGTCATGTACCAGTGGCT 8640

8641 GCAACACTGAAGAAAATGCCTCTCCCTTCCCTGGAAGCTGTTAGCTGCCTACAAATCCCC 8700

8701 AGGGAGGAGTGGGTCCTCATTTGACTCCCCACTCCTTCAACACAACAACTTCTCTTGAGC 8760

8761 TCCACCCCAGCCCCAGGTCTTTAAATAGTTGTCCACATGGAAGAGCGAGCCCTTGAGTGA 8820

8821 CCTGTGGCCTTAGGTAATGGTGGAGTGTCATGTAGGGTTGATGACTGTCACAAGTGGACC 8880

8881 AGTTGTCTGTGGGGAGTTGATGGTGCTGAGGCTGTATACATGGAGACAGAGCTCCATTTG 8940

8941 GCTGACAATCCAAGATAGCTGAAAAAAAATAGTTTAAATCTGGGGCTGTGCCTCAATGGG 9000

9001 AAGAACATGTGTCCAGTGTGCACAGAGTCCTGGGTACC 9038
