# Supplementary material for: Expression of a Chimeric Antigen Receptor in Multiple Leukocyte Lineages in Transgenic Mice
Source: PLoS One. 2015 Oct 27;10(10):e0140543. doi: 10.1371/journal.pone.0140543 (PMC4624721; doi:10.1371/journal.pone.0140543)

S2 Fig

A.

AGTGCCCCAT TTTCTTCCTG GGACTTCTGA CTAGCAGGTG AAAGTTAGAG C**CTAGCAGTT**
CCTGTTGAGA TAGTATAAAG GCTATTTACT TAATTCTTTG TGGTTTGAGG AAGAGGTGCT
AAGTGTCAGA AACTGTAGTT TATGGCCTCA GAGGATCTCA GAAGGCAGGT CAGCTACAGT
AGCACAGTAA CTTAGATAAG TAAACTTCAT TATACAGCAT CCCTAAATAT CTCATAAGAC
AAAGTCTTAC CCCCAATCTC GTAAGACCAA GTTTTACCAT CCGCTGAAAC TCTTCTCCCT
CCGCTGCTTC AAGAAGCACT CCACCATGGG GCTGCTCCCC ACCACCTCCC CACAGAAAAG
AATACTGATG TTTAGCAACT GACATAGATC CAACTACTTT TTAAAATATT TGTTTATACC
CATAGTGGTA CACTACTCTC AGTCTTAATC TCTCCAGTAG ACTGTAAATT AAAACAGAGG
TGTAAGCTAC CTCCACAGGG TACTAAGAAC ATATGATGGC TGAGTGTTAA GCCTTAAATA
AGACATTTAT ACCATCCTGT CTTTAGTTCA CAGAACACTG AATATAGAAT TGAAATGGAA
AAGGATGTAA GAGCTGATAT GGGGAGAAGG CTATAAAGTG ACATCTCCTG GTAAGACACA
GCTATTTCAA TCACAAACTC AAAGCAACCA AAGCTAACTT CACTAAGTAT GCATGAAATG
GAACTGTTAA CAGTCTAGTA TGTCTAGATG AGGATCTCAG AAGGTTCTGT TCTTTACTAG
TGGACTGATA GATTTAGGGG AAAGGACTTT TTGTGTCCAT TGATAATTCT ACCAGGTTCC
AATGTATAGT TCTAATTCAA TGGGTAACAG AGATGAATAA ATAAAATGCA TTACCTATAA
AAATTAGATT TTTATTATCT TAAATAAAAA TGAAATCATG AGATTTGCAA GAA**AATGAAA**
TGACCTGGTA ATCATTATAT TAAGCAAAGT TAGTCAAACT TAGAGAAATA CTACATATTT
TCCCTTATAG AGTCTAGAGT TAAGTATAGA TATGTACATG TAAGCTTGGC TGCAGGTCGA
AATTCGAATC TAGAAACCTA GTGGGCGCTC TCCAGCTGCC TCTCGTTTTT GTTTTGTTTT
CACTGCCTGG CCTTCCTACC CTAACTGCGT GCAAAGAAAA CAAATTGTTA CCCGGTTTAG
AGAAGAGGGA GAGGACGCGT AGGAAGTCAC AGACGCCAAA AATAGG**GGAT AGAAATTGTC**
CTCGCCTGTA AGGTTGCAAA ACGGGAGGGT TTTCAGGTTG TGCCCACCCC TACCCATATC
- - - - - - - - Seven copies of VAV-CAR transgene - - - - - - - - - -
AAGTGTCAGA AACTGTAGTT TATGGCCTCA GAGGATCTCA GAAGGCAGGT CAGCTACAGT
AGCACAGTAA CTTAGATAAG TAAACTTCAT TATACAGCAT CCCTAAATAT CTCATAAGAC
AAAGTCTTAC CCCCAATCTC GTAAGACCAA GTTTTACCAT CCGCTGAAAC TCTTCTCCCT
CCGCTGCTTC AAGAAGCACT CCACCATGGG GCTGCTCCCC ACCACCTCCC CACAGAAAAG
AATACTGATG TTTAGCAACT GACATAGATC CAACTACTTT TTAAAATATT TGTTTATACC
CATAGTGGTA CACTACTCTC AGTCTTAATC TCTCCAGTAG ACTGTAAATT AAAACAGAGG
TGTAAGCTAC CTCCACAGGG TACTAAGAAC ATATGATGGC TGAGTGTTAA GCCTTAAATA
AGACATTTAT ACCATCCTGT CTTTAGTTCA CAGAACACTG AATATAGAAT TGAAATGGAA
AAGGATGTAA GAGCTGATAT GGGGAGAAGG CTATAAAGTG ACATCTCCTG GTAAGACACA
GCTATTTCAA TCACAAACTC AAAGCAACCA AAGCTAACTT CACTAAGTAT GCATGAAATG
GAACTGTTAA CAGTCTAGTA TGTCTAGATG AGGATCTCAG AAGGTTCTGT TCTTTACTAG
TGGACTGATA GATTTAGGGG AAAGGACTTT TTGTGTCCAT TGATAATTCT ACCAGGTTCC
AATGTATAGT TCTAATTCAA TGGGTAACAG AGATGAATAA ATAAAATGCA TTACCTATAA
AAATTAGATT TTTATTATCT TAAATAAAAA TGAAATCATG AGATTTGCAA GAAAATGAAA
TGACCTGGTA ATCATTATAT TAAGCAAAGT TAGTCAAACT TAGAGAAATA CTACATATTT
TCCCTTATAG AGTCTAGAGT TAAGTATAGA TATGTACATG TGATGACACT AGAAAGGGAT
CATGGGAGGG GAGGAAGGCA T**CATAAGGAG AGAGAAGCAA GT**AAGAGAAT ACAAATGACC
TGAAGACAGA AAAGTTGCTG CTGTTACCGA GGTGTATTCT ACTTCTTTAC ACTGCATGTA

B.

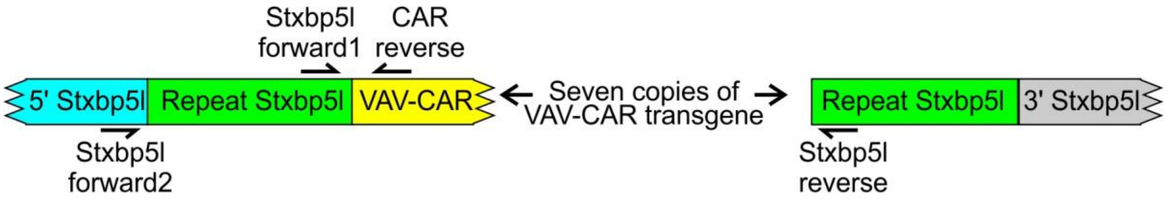

Supplement: S2 Fig — (Fig A) Blue highlight = upstream chromosome; Grey highlight = downstream chromosome; Yellow highlight = 5’ end of vav-CAR transgene; Green highlight = duplicated section of Stxbp5l gene, flanking seven copies of transgene insertion. Bold underlined text = genotyping primer sequences. (Fig B) Schematic representation of the insertion point with PCR primer positions indicated. (PDF) [file pone.0140543.s002.pdf]
